# Evaluation of the initial rollout of the physical activity referral standards policy in Scotland: a qualitative study

**DOI:** 10.1136/bmjopen-2024-089723

**Published:** 2025-01-23

**Authors:** Coral L Hanson, Sheona Mchale, Lis Neubeck, Nadine Dougall, Paul Kelly

**Affiliations:** 1 Centre for Cardiovascular Health, School of Health and Social Care, Edinburgh Napier University, Edinburgh, UK; 2 Sydney Nursing School, Charles Perkins Centre, University of Sydney, Sydney, New South Wales, Australia; 3 Centre for Mental Health Practice, Policy and Law, School of Health and Social Care, Edinburgh Napier University, Edinburgh, UK; 4 Institute for Sport, Physical Education and Health Sciences, Physical Activity for Health Research Centre, University of Edinburgh, Edinburgh, UK

**Keywords:** Health policy, PUBLIC HEALTH, QUALITATIVE RESEARCH, Exercise

## Abstract

**Abstract:**

**Objectives:**

Physical activity referral schemes (PARS) allow healthcare professionals to refer patients for physical activity support. Evidence of effectiveness is equivocal. Public Health Scotland has developed ‘physical activity referral standards’ that aim to enhance quality, reduce variability in design and delivery and build further evidence of what works. This study evaluated stakeholder perspectives on the initial reach, adoption, implementation and effectiveness of the standards.

**Design:**

A qualitative study using individual, online, semistructured interviews to explore stakeholder awareness and willingness to use the standards. We analysed data using the framework method within the context of the RE-AIM (Reach, Effectiveness, Adoption, Implementation and Maintenance) framework.

**Setting:**

Data were collected across 28 local authorities in rural and urban areas of Scotland between December 2022 and June 2023.

**Participants:**

73 stakeholders, including scheme managers (n=34), senior managers from provider organisations (n=9), healthcare professionals (n=19) (general practitioners, nurses, occupational therapists and physiotherapists) and policy stakeholders (n=11).

**Results:**

72.6% of stakeholders were aware of the physical activity referral standards, and they were widely welcomed. Healthcare professionals were the least informed. Participants appeared willing to adopt the standards, and stakeholders reported using them to help with service planning, audit delivery processes, identify service gaps, inform monitoring and evaluation plans and understand and communicate the roles and responsibilities of different partners. Barriers to implementation included lack of healthcare professional awareness, funding and workforce capacity. Views about the minimum dataset (suggested essential or desirable data fields to be collected for monitoring and evaluation) contained in the standards were divided. Some thought it useful, but others considered it onerous or aspirational, and it was unclear whether all service delivery stakeholders would have the resources or capacity to collect and analyse the data.

**Conclusions:**

The delivery of the standards could be enhanced by a comprehensive communication strategy and by addressing the lack of funding, workforce delivery capacity and skills/capacity required to collect and interpret the proposed minimum national dataset.

**STRENGTHS AND LIMITATIONS OF THIS STUDY**

- This study included a large sample size for a qualitative study, recruitment from 88% of Scottish administrative areas and multidisciplinary representation.
- The breadth of recruitment ensured the capture of wide-ranging views, experiences and insights from rural and urban areas.
- Data were analysed within the RE-AIM framework structure using rigorous, qualitative approaches.
- The relatively short timeframe between the physical activity referral standards launch and the study means that policy implementation has not been fully explored, and it was too early to assess maintenance.
- The specific public health structures and context in Scotland may limit applicability to other countries.

## Introduction

 Physical inactivity increases the risk of conditions such as cardiovascular disease, type 2 diabetes and some cancers.^1^ Globally, however, many people are insufficiently active to reduce health risks.^2^ Physical activity referral schemes (PARS), also known as physical activity prescriptions or exercise referral schemes, are important interventions that are included in national physical activity pathways and recommendations.^3 4^ Healthcare professionals (HCPs) can use them to encourage patients with a range of non-communicable diseases to be physically active.^5^ In the UK, PARS involve HCPs referring patients with a range of health conditions to leisure organisations, who provide individualised assessments interspersed with approximately 10–16 weeks of supervised or independent activity.^6^ Systematic reviews have demonstrated modest positive impacts on physical activity (PA) but suggest that effectiveness and cost-effectiveness may be sensitive to small design and delivery differences.^7 8^ The variety and complexity of PARS are well acknowledged^9^ and uncertainty exists about what predicts uptake (how many of those referred initially participate) and adherence (of those who start, how many continue to participate, in what and for how long).^9^,^11^ A lack of standardised reporting of what is delivered, how and by whom, definitions of uptake and adherence, and performance measures mean that it is difficult to assess what elements of schemes are most likely to result in improved outcomes and define what works best.^9^,^12^ Therefore, strategies that recognise the variability of scheme delivery but support the standardisation of monitoring, evaluation and reporting may help to address these issues at local and national levels.

The first UK PARS National Quality Assurance Framework was published in 2001,^13^ but this did not recommend standardised practice or evaluation criteria, and no attempt was made to assess implementation. In 2010, the British Heart Foundation National Centre for Physical Activity and Health published ‘*A Tool Kit for the Design, Implementation & Evaluation of Exercise Referral Schemes*’.^14^ This suggested the need for PARS to create logic models and conduct process and outcome evaluations. The toolkit highlighted validated questionnaires previously used in PARS literature without specific usage preferences. In 2014, the UK National Institute for Health and Care Excellence recommended that PARS providers and researchers should focus on understanding how scheme characteristics (eg, setting, duration and intensity, target groups, referral mechanisms and provider qualifications) and components (eg, behavioural support techniques and PA choices) influence effectiveness.^15^

In 2022, Public Health Scotland (PHS) published national physical activity referral standards (the standards)^16^ after extensive consultation with stakeholders. This involved the development of a logic model (online supplemental file 1) that focused on the wider PARS system. The standards aim to enhance PARS quality, reduce variability in design and delivery and build further evidence of what works.^16^ There are six core components: (1) partnership working, (2) local delivery models, (3) learning and workforce development, (4) data systems, (5) monitoring and evaluation (including use of the PARS taxonomy^10^ to report design and delivery and a national minimum dataset, which included suggested essential or desirable data fields to be collected for monitoring and evaluation such as demographics, referral numbers, uptake and adherence) and (6) sharing learning and good practice.^16^

There are notable gaps between the existence of policies to promote PA and their operational status.^17^ Policies such as the standards often lack tangible steps to facilitate adoption, and little is known about implementation at national or local level, largely due to a lack of formal systems or reporting procedures.^18^ The RE-AIM (Reach, Effectiveness, Adoption, Implementation and Maintenance) framework is a commonly used planning and evaluation framework across public health, behaviour science and implementation science.^19^ This study aimed to understand the early policy delivery process for the standards to inform ongoing implementation. We used RE-AIM as a framework to situate our findings and help explain whether and/or how the standards are used.

## Methods

### Study design

This study was an observational evaluation, situated within a constructivist, interpretative epistemological approach, of a national policy rollout using qualitative methods. Individual semistructured interviews were used to explore knowledge of, and perceptions about, the standards in different PARS stakeholder groups. This study was the first part of a larger project that includes using mixed-methods to explore the design and delivery of PARS, monitoring and evaluation strategies, and participants’ views of how schemes should be monitored and evaluated. A favourable ethical opinion was given by Edinburgh Napier University School of Health and Social Care Research Integrity Committee (REF: SHSC2912253).

### Patient and public involvement

The study is part of a wider project with a steering committee that includes five PARS participants (people who had been referred to a PARS in Scotland). The protocol and lay summary of results for the study were shared with PARS participants prior to in-person steering group meetings, then discussed in detail and agreed upon during meetings.

### Sample and researchers

A purposive sample of Scottish PARS stakeholders, including senior managers of PARS provider organisations (responsible for strategic planning for health and well-being activities), PARS coordinators (responsible for managing the day-to-day delivery of schemes), HCPs able to refer to PARS and those involved in developing and/or implementing PA policy (working in government, national sporting bodies and Scottish NHS health boards). Within these groups, there were no exclusion criteria. Email invitations were distributed via established networks (PHS national PARS development group, Movement for Health Coalition of Scottish health charities, Community Leisure UK and Faculty of Sport and Exercise Medicine). Additionally, participating PARS advertised the study to referring HCPs, and we undertook internet searches to identify PARS. The sample included stakeholders who had been involved in the standards development. We used snowball sampling to increase numbers. Our sample included four stakeholder groups, and the sample size was based on the potential to achieve data saturation within each group at between 9 and 17 interviews, based on systematic review recommendations by Hennink and Kaiser.^20^ PARS coordinators were an exception, where a large sample size was required due to the variety of different approaches to scheme delivery. Individuals were sent an online link in recruitment emails that provided access to a participant information sheet, privacy notice, and consent form. Participation was voluntary, and individuals gave online informed consent via the secure NOVI survey system (NOVI Survey, Cambridge, Massachusetts, USA) to register for the study. Researchers then contacted participants via email or telephone to arrange interviews. One week before interviews took place, participants were sent a link to the standards so that they could read them if they wished to.

The researchers, females (n=4) and male (n=1), were university-based with PhDs and experience in PARS (CLH and SM) and evaluation and implementation science (PK, CLH, LN, ND and SM). Experienced qualitative researchers were responsible for data collection (SM and CLH) and analysis (SM, CLH, LN and PK). Two authors (PK and CLH) were involved in the development of the standards. We acknowledge that this experience may have influenced their outlook and approach to the study. To mitigate potential biases, SM was the lead analyst to help maintain a more objective and independent perspective.

### Data collection

Interview guides contained open questions about knowledge of and perceptions about the potential use of the standards (online supplemental file 2). Data were collected by two researchers (SM and CLH) in private via individual semistructured interviews using Microsoft Teams (Microsoft Corporation, Bellevue, Washington, USA) between December 2022 and July 2023. SM and CLH had prior relationships with some participants due to previous PARS studies and PHS events organised to develop the standards. Researchers kept field notes and reflective diaries to record contextual information about interaction quality and potential biases.

### Data analysis

Interviews were transcribed verbatim, anonymised and imported into NVivo20 (QSR International,Melbourne, Australia), a data analysis organisation tool. Data were analysed using the framework method.^21^ During the familiarisation phase, SM and CLH checked transcripts with audio recordings for accuracy. They independently created inductive open codes (n=97) for three transcripts in each stakeholder group. In the identification phase, they grouped open codes into 12 initial inductive themes (awareness, usefulness, the tiered model of PA interventions, local delivery contexts, cost, inequalities, sharing good practice, workforce issues, data systems, monitoring/evaluation, communication and partnerships). Initial themes were mapped to the reach, adoption and implementation elements of the RE-AIM framework^19 22^ to structure the findings. This initial framework was applied as a tool for interpretation, as opposed to theme development, and presented to the other researchers during a data workshop. After a critical discussion, the inductive themes were reorganised within the RE-AIM framework to include effectiveness (online supplemental file 3). During indexing, SM coded 10 more transcripts, which CLH checked for coding accuracy by reading code content and using NVIVO20 deductive word searching. SM then coded all remaining transcripts. Analysis took place alongside data collection to gauge when no new meaning of codes or themes and their relationships were apparent. NVivo framework matrices were used during the charting phase (an example can be found in online supplemental file 4). Researchers explored and rearranged the data within the inductive themes and compared across cases to develop explanatory summaries. Prior to the final analysis, the framework was reviewed, and the analysis mapped to the reach, effectiveness, adoption and implementation elements of RE-AIM.

Where data were quantifiable (eg, awareness of the standards), we collated results in a Microsoft Excel spreadsheet (Microsoft Corporation, St. Redmond, Washington, USA).

## Results

**Figure 1:**
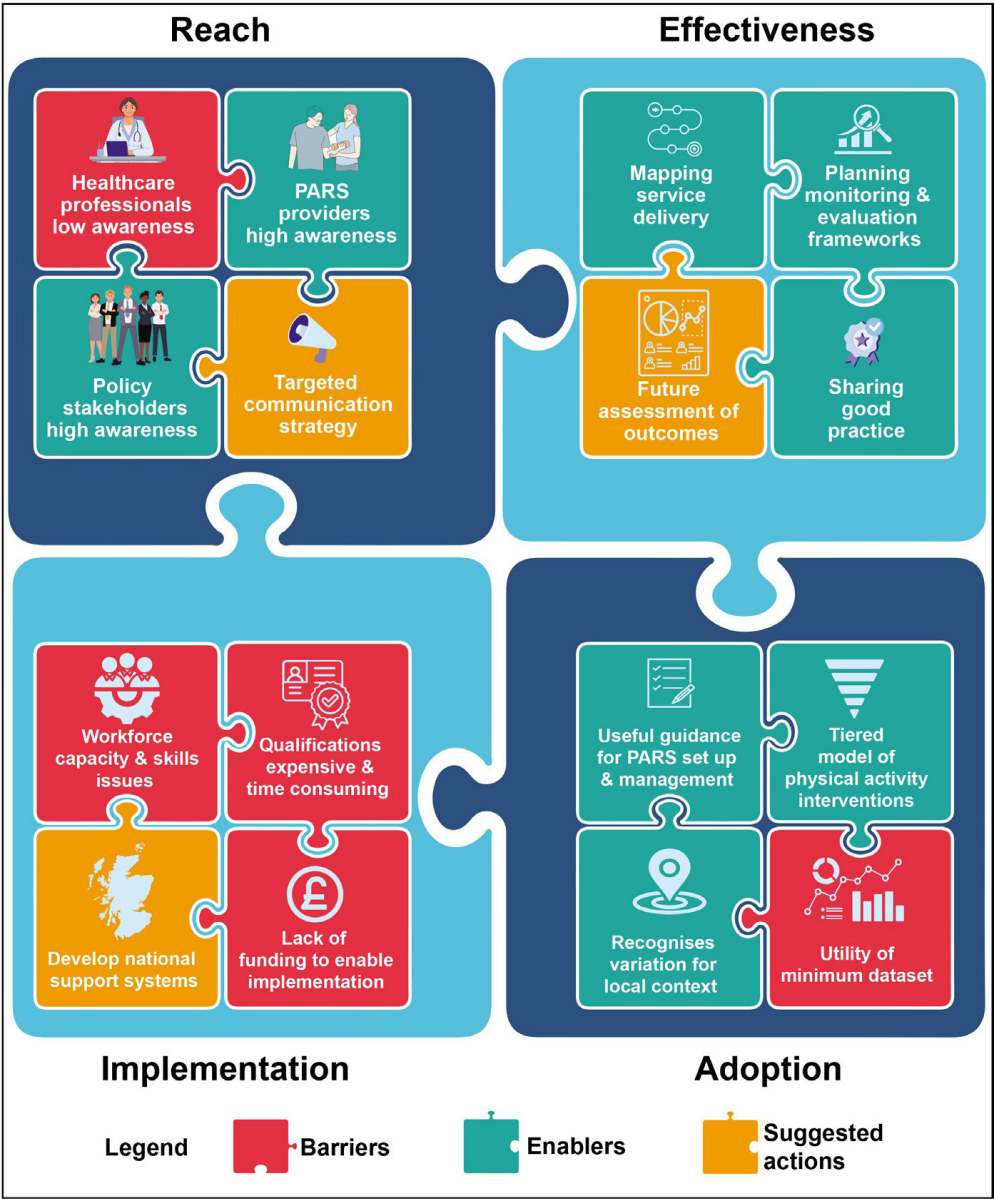
Public Health Scotland physical activity referral standards policy rollout factors.

95 people were invited to participate. Eight did not respond, seven declined to take part (one due to maternity leave, four due to lack of time and two with no reason), 75 consented and 73 (97.3%) completed interviews. We were unable to arrange suitable interview times for those who consented but did not take part. Most participants were female (n=48, 65.6%), and the largest stakeholder group was PARS coordinators (n=34, 46.6%), compared with PARS provider senior managers (n=9, 12.3%), HCPs (n=19, 26.0%) (GPs (n=6), nurses (n=2), occupational therapists (n=1) and physiotherapists (n=10)) and policy stakeholders (n=11, 15.1%). The participants were drawn from 28 of the 32 local authority areas in Scotland, indicating a good spread of stakeholder groups and geographic representation. Interviews ranged from 48 to 90 min. Findings were mapped within the reach, effectiveness, adoption and implementation elements of RE-AIM (figure 1). Indicative quotes are presented in tables at the end of each section.

### Reach

We defined reach as awareness of the standards. We explored what level of knowledge about standards content was required for different stakeholder groups to be able to implement them. Overall, 72.6% (n=53) of stakeholders were aware that the standards existed prior to the study, but this varied by stakeholder group: PARS provider senior managers (n=8/9, 88.9%), PARS coordinators (n=27/34, 79.9%), policy stakeholders (n=11/11, 100%) and HCPs (n=7/18, 36.8%). However, the level of knowledge about content varied, with policy stakeholders and PARS coordinators being able to describe/discuss more components without prompting during interviews.

PARS provider awareness had been raised via national or local public health meetings or cascading within organisations. Providers did not think that many HCPs were aware of the standards and highlighted the need for PHS to ensure understanding and buy-in by all stakeholders. Policy stakeholders reported a soft launch of the standards in 2022 but stated that a formal launch was still required. Prior to this, policy stakeholders suggested that time should be taken to identify target groups who should be aware of the standards and that messaging should be developed for each group on a ‘need to know’ basis. This was supported by HCPs, who stated that they received a large amount of information per week and would prefer to only be given information relevant to their practice (eg, who, how and where to refer and how to record referrals on primary care systems). Simply circulating the standards was not considered appropriate as there were concerns that they might not be read. Instead, HCPs suggested alternative methods of communication, such as a concise overview from local PARS providers or summarised highlights via an infographic. Indicative quotes are presented in table 1.

**Table 1:** Reach of the Public Health Scotlandphysical activity referral standards

Stakeholder groups	Indicative quotes
Senior managers within PARS provider organisations	‘Whenever I have sat down with healthcare professionals very few of them are aware of the standards…I would probably say actually, do they need to know? Probably not. Not everyone certainly needs to know’. (Participant 75)‘They (Public Health Scotland) have to make sure that everybody at every level understands them and has buy-in with them’. (Participant 72)
PARS coordinators	‘I was involved in a few of the meetings, just, kind of giving my voice from leisure at the very, very beginning’. (Participant 1)‘Part of a meeting with Public Health (Scotland), we discussed the standards and then it was part of…I attended a focus group to discuss how we implement them’. (Participant 9)
Policy stakeholders	‘They were soft launched in February 2022, but there wasn't really a song and dance about them, …and I think it’s probably taken those that were aware of them at the time probably until now to even just start getting their head around them. There needs to be more of a formal launch’. (Participant 6)‘I found out about the Standards… originally when they were being developed. So I met with Public Health Scotland. They…were seeking views and opinions of individuals and they knew the work that we were doing in the space, and they felt it would be really important to engage us and get our perspective and our experience and expertise’. (Participant 54)
Healthcare professionals	‘An infographic is usually pretty good because, you know, it draws your eyes to certain things. I think, the things that don’t work are lengthy documents that are all text…because we’re very time precious in the service’. (Participant 53)‘So, a shortened version with the main things, and for us it would be… about the tiers, about the referral processes and,…maybe how they do make decisions about where people go…And maybe something around about,…the systems they use for referral’. (Participant 73)

PARSphysical activity referral schemes

### Effectiveness

We defined effectiveness as what PARS outcomes result from actions taken as a consequence of the publication of the standards. At this early stage of use, it was difficult to assess whether or how they had changed practice and what difference this might make to outcomes. Indeed, most participants were unable to describe existing outcomes, and the standards were seen as a facilitator to create systems that would allow for an understanding of these. Participants reported that they were using the standards to map current delivery and identify gaps. They commented that this supported a systems approach to targeting inequalities. Some PARS providers were collecting postcode data to assess programme reach and most acknowledged the need to target population groups currently underserved by their schemes. Policy and HCP stakeholders emphasised the importance of identifying the needs of local communities to avoid exacerbation of existing inequalities.

PARS providers reported that they were using the standards as a guide to planning monitoring and evaluation frameworks to collect data about participant demographics, engagement and, in some cases, resultant behaviour change. It was still too early to understand whether the standards have provided impetus for change that will result in increased access by target groups, such as those living in areas of socioeconomic deprivation. To understand whether change was needed, stakeholders stated that they required information about scheme performance, and PARS providers expressed a need for further guidance on how to effectively consider and address equality issues.

Sharing good practices was considered essential to supporting learning about PARS effectiveness. Participants reported that good practice was routinely shared at a local level with funders, other local partners and communities via newsletters, case studies, infographics, reports and social media. PARS providers also shared good practices via forums such as Community Leisure UK but felt that a more coordinated approach led by PHS was required. Suggestions included a PHS national forum to showcase what works well, the need to recognise different solutions for local area requirements (eg, rural vs urban) and a national repository of resources that would help to improve schemes. Indicative quotes are presented in table 2.

**Table 2:** Effectiveness of the Public Health Scotland physical activity referral standards

Stakeholder groups	Indicative quotes
Senior managers within PARS provider organisations	‘in the guidelines it’s talking a lot about equalities impact assessment and …we have not done equalities impact assessments.…although I feel confident that we're doing a lot around this issue. It’s just a slightly different approach. But one thing I thought was maybe missing from that was the guidance around how to do it’. (Participant 17)‘There’s plans to use it (the standards), for example, around SIMD, and around things like ethnicity, to ensure that we are ensuring that marginalised groups are included within our services’. (Participant 62)
PARS coordinators	‘…these are the guidelines and then it is pretty much up to us to look at our programme and maybe make some adjustments to that. So, the big adjustment was adding that International Physical Activity Questionnaire. Before we had another activity questionnaire’. (Participant 30)‘So somewhere like a national (private web page) that organisations can just post something up to. It doesn’t need to be, a big meaty chunky bit of work, it could just be, oh this happened today, and we found a real good learning experience from it…something that’s easy to access that we could all dip in to…I mean, there might just be a shared channel on Teams or somewhere that people can drop documents or stories of interest’. (Participant 7)
Policy stakeholders	‘It’s not enough to just say inequalities underpin everything that we do. But you know, where’s our data around inequalities? Where’s our specific actions that are looking at particular groups and what we are doing there?’ (Participant 19)‘But we’re not comparing apples with apples here, you know, the demographics of our country are so varied that the consistency is the long term health condition aspect of it…And then we’ve got our rural authorities as well, so…for me, the whole premise of it is being person centred. So, I would hope that that is what drives the differences within some of the schemes’. (Participant 68)‘I think it’s more about having a forum by which these things can be shared and therefore probably Public Health Scotland perhaps owning that forum’. (Participant 19)
Healthcare professionals	‘Reading the Standards, I'm not aware of anywhere that’s offering true Tier two physical activity referral where we refer to, they might be (level 4) qualified, but…I don't see where the health behaviour change is coming into that at all, which I think is really key actually’. (Participant 18)‘I think the big one for me is about inequalities. Because with all social prescribing, my biggest worry, and my biggest problem with it is worsening health inequalities by getting very healthy people even healthier. Umm and….You know, a scheme might look great by itself on itself, but actually unless you've got that inequality data you don't know whether they're worsening things or not, and I you know, and I look at our own data for the (PARS) and we haven't, we haven't done work properly on that yet’. (Participant 38)

PARSphysical activity referral schemesSIMDScottish Index of Mulitple Deprivation

### Adoption

We defined adoption as understanding what elements of the standards influence decisions about use and in what settings. All participants agreed that the standards provided useful guidance for setting up and managing PARS. They recognised that the standards did not provide a blueprint for scheme structure and that models would differ depending on context. The most discussed element of the standards was the utility of the proposed minimum national data set for future evaluation and the data systems that were required to collect this. While the minimum dataset was considered important, not all providers intended to collect all elements of it. For PARS providers, this was because it did not align with funders’ reporting requirements or because of a perceived lack of value in the measures. Additionally, some HCPs and PARS providers expressed apprehension about the extent of measures and raised questions about the benefits of collecting such data. Other HCPs questioned why the adoption of the standards was not mandatory across Scotland to standardise approaches.

The standards contained a tiered model of PA interventions. This was a conceptual diagram indicating differing levels of PA intervention, target audiences, behaviour change approaches and appropriate support for long-term conditions at each level. It was considered to be helpful in identifying different referral/signposting options for HCPs and delivery modalities for PARS providers. Despite some HCPs expressing concerns that the tiered model pyramid was inverted, they acknowledged that it was easy to understand in relation to other health pathway guidance documents because it used a similar numbered approach. PARS providers were not always clear about which tier the interventions that they offered fitted into, and this was problematic as the standards were aimed at tier 2 schemes. HCPs felt that the current PARS did not necessarily fulfil all criteria for a tier 2 scheme (eg, they lacked behaviour change techniques). Many areas reported the intention to map all PA interventions against the tiered model to provide HCPs with more comprehensive PA options to discuss with patients, potentially leading to more tailored referrals. This was an unexpected but potentially valuable element of standards adoption and one that HCPs welcomed. Indicative quotes are presented in table 3.

**Table 3:** Adoption of the Public Health Scotland physical activity referral standards

Stakeholder groups	Indicative quotes
Senior managers within PARS provider organisations	‘But the physical activity referral standards ask for too much, that’s the one negative side. And I understand why they ask that, and in an ideal world it would be great to have all this information, but it’s a huge amount of information’. (Participant 62)‘I think if there is a national data set then it means that potentially you could draw some pretty useful conclusions across the board’. (Participant 75)
PARS coordinators	‘…So, you're asking me for all of this information. So, what are you going to do with it? And what difference is it going to make? Sorry, that’s probably quite a lot in there, but I think it’s…complex. I'm not saying it’s not valuable, it absolutely is’. (Participant 14)‘There’s been a bit of discussion around the leisure trust and an official referral pathway, all we’re seeing is tier two…Where there’s a little bit of crossover is the tier one pathway because, for instance, there’s a few of us already involved in prehab’. (Participant 36)‘I mean we don't use all of these things. I think our questionnaires are tailored very specifically to report back to our funders’. (Participant 64)
Policy stakeholders	‘We have used the Standards as almost a guidance document for sense checking’. (Participant 11)‘I do actually like the tiered system…and we have been using it …linking our programmes to their referrals as well…we've struggled a little bit with some of the tiers’. (Participant 23)
Healthcare professionals	‘So normally when we look at pyramids, they are the other way up, and I would have clarified tier 1 as tier 5 and then all the way up’. (Participant 48)‘…This is an awful lot of things to gather about people. Are we making this as accessible as we’d like to make it?’ (Participant 46)

PARSphysical activity referral schemes

### Implementation

We defined implementation as understanding the barriers and facilitators to actioning the standards. All stakeholders were positive about the value of PARS, but responses reflected the challenges in implementing schemes.

PARS providers reported that although the standards could help decision-makers understand roles and responsibilities within PARS, they did not equate to funding. As resources were stretched in most areas, cost was a potential barrier to implementation. This was because where funding did exist, stakeholders reported that it included the cost of PARS delivery staff but not management. Despite this, some believed that implementing the standards might help secure future funding.

Where providers were planning to collect the minimum national dataset, they were concerned that they did not have the skills or capacity to interpret it. Some PARS providers had existing data systems that allowed them to collect data at a local level, but these did not necessarily help to provide evaluation reports. Some suggested that a national database and dashboard to collate and provide evaluation feedback would help implementation.

Workforce capacity was reported to be a key barrier in implementing the standards. Providers stated that qualified and experienced staff had left during COVID-19. Referral numbers were reported to be increasing rapidly postpandemic, and there were insufficient staff to deal with the numbers. Recruiting appropriately qualified staff was extremely challenging, and new recruits required time for training. Recognised qualifications were reported to be expensive in a financially difficult landscape. Additionally, ongoing professional development for current staff was perceived to be challenging to support. Membership of the Chartered Institute for the Management of Sport and Physical Activity was not valued by PARS staff, and there were concerns about the cost-effectiveness of accreditation and additional learning components. This created conflict with the learning and workforce development element of the standards.

PARS providers had differing opinions about whether implementing the standards would help to build partnerships but felt that they provided focus and enhanced credibility, especially in health collaborations, as this was considered to be limited at present. PARS coordinators believed that standards compliance would demonstrate commitment to responsibility and quality. Participants from all stakeholder groups questioned whether the standards could lead to a future quality assurance scheme, although they acknowledged that implementing and maintaining such a scheme would be complex and require funding. Indicative quotes are presented in table 4.

**Table 4:** Implementation of the Public Health Scotland physical activity referral standards

Stakeholder groups	Indicative quotes
Senior managers within PARS provider organisations	‘Workforce is a big risk for us…ensuring that we've got the right staff to deliver, the qualifications to deliver, and the cost of qualifications…’ (Participant 17)‘It is all very well to have the standards and the guidance; they don’t equate to money’. (Participant 51)‘We see it as being really critical to have standards in place for loads of different reasons. Quality assurance…one of the biggest barriers that we face is being able to assure the healthcare professionals that we work with that the service that we could offer is quality’. (Participant 75)
PARS coordinators	’You know, if there was two or three of me…or if there were more guys on the ground…and they had more time to actually do the monitoring and recording that we would want, then…yeah. So… I think my whole conversation is probably based around capacity and funding’. (Participant 10)‘The only thing I would say about that referral guidelines, is it makes for an expensive programme’. (Participant 22)‘In an ideal world Public Health Scotland or the Scottish government could create us a portal or something that the guys could just use to record all this information…’ (Participant 10)’We've got some…good experience within that workforce, that is if we were to lose one or two it could affect our output. So, it’s about where’s the next one coming through? Where can we get our next level four instructor, to gain that experience and knowledge. So, workforce and succession planning is probably one of the biggest challenges’. (Participant 26)‘I think for us looking at how we support them around CPD is going to be really important’. (Participant 40)
Policy stakeholders	‘There’s huge pressures on leisure facilities in terms of income, generations, staffing, etc…what we really need to do is…we need to bring in some more investment so we can actually give them the protected time they need to deliver the level of scheme that they want to deliver’. (Participant 21)‘At the moment there’s nowhere to collate all this. So even if it does happen, there’s nowhere for it to go. There needs to be some sort of strategic leadership in Scotland at a government level to say that you know this is really important that this is done’. (Participant 6)‘All the facilities saw the value of the indicators within the minimum data set, but it just kept coming back to the capacity to collect that data and then actually once you've got the data, what you do with it in terms of processing it…’ (Participants 21)
Healthcare professionals	‘…none of us are individually going to go and check whether they fulfil those sets of standards. So, if you're saying there’s a set of standards, then they probably …it would be relevant to us if there was a check. You know a kind of a seal of approval…’ (Participant 38)‘…it allows us to ensure that the level of specific, condition specific training is there, but also it allows us to strengthen our partnership’ (Participant 55)

PARSphysical activity referral schemes

## Discussion

### Principal findings

We analysed stakeholder views about the early delivery of the PHS physical activity referral standards, considering the reach, adoption, effectiveness and implementation elements of the RE-AIM framework. 73% of participants were aware of the standards (reach), although this varied between stakeholder groups, and HCPs were the least informed. The standards were a widely welcomed policy initiative, and stakeholders appeared engaged and willing to adopt and implement them. Barriers to doing so included funding and workforce capacity. In this early phase, the standards were reported to be informing and changing practice. They were being used to help service planning as a service audit tool to identify delivery processes and service gaps, inform monitoring and evaluation plans and help understand and communicate the roles and responsibilities of different partners. While some thought it useful, the national dataset was seen as onerous or aspirational by others, and it was not clear that all service delivery stakeholders had the resources or capacity to collect and analyse the data. The tiered model was considered useful because it illustrated links to other related services such as social prescribing.

### Possible explanations for results

The successes of the early reach and adoption phases of the standards delivery may be linked to two factors. First, Scotland has a relatively small population of 5.4 million and only 32 local government areas.^23^ Although policies formulated at the national level face challenges when delivered at the subnational level,^24^ this is possibly less of a problem for smaller nations where cohesion is easier. However, Scottish administrative areas have very different geographical and demographic profiles, and population density ranges from <10 to >3500 residents per km^2^.^2^,^23^ This creates challenges. To ensure successful adoption and implementation, policies must be adaptable to fit differing contexts and practices.^25^,^27^ The standards recognise the need for local variation and highlight that delivery should fit with local outcomes, improvement plans and public health priorities.^16^ Participants in our study were clear that the standards were not a PARS blueprint, and recognition of local contexts contributed to the willingness to adopt and implement the policy.

The second facilitating factor is the strong existing links and networks between Scottish PA promotion stakeholders. The collaborative nature of the standards development included several in-person and online workshops with key stakeholders (including two study authors, CLH and PK) over 2 years. Coproduction improves multisector alignment and policy feasibility,^28^ and this approach likely encouraged engagement in the initial policy rollout. Despite informal dissemination, many service planners and providers were aware of the standards due to the development process. The involvement of few HCPs in the development may explain the lower reach in this group. The need for a comprehensive communications strategy and subsequent partnership working has been identified as essential to successfully disseminate, implement and further develop health policies.^29^ We highlight the need to improve HCP awareness of the standards. Additionally, continuing national coordination of stakeholder engagement is needed to explore the successes and challenges of adoption and implementation and ensure policy adaptations where required. This is important because the next phase of policy embedding must demonstrate success, drive cross-organisational working and refresh the coalition of external support.^30^

### Comparisons with other studies

We identified similar adoption and implementation challenges across organisations in the study. These were communication, a lack of funding, delivery capacity, skills, resources and time and questions about the appropriateness of the minimum data set. Our findings are aligned with other PA policy studies, which identified the need for better communication,^27 31^ funding,^27 31 32^ improved capacity^31 32^ and a focus on appropriate outcome measures.^32^ Funding is perhaps the most important, as it helps address other barriers. Pressure on UK public health and local government funding means that policies, such as the standards, aimed at reducing health inequalities are potentially being set up to fail.^33^ Non-statutory services such as PARS are often end receivers of public funding and so are particularly vulnerable to funding cuts. If future funding is not available, there is a high risk that the policy will fail.

There are limited adequate evaluations of PA policy implementation.^34 35^ We identified international surveys about the adoption and implementation of health-enhancing PA across sectors,^36 37^ audits of national PA policy documents,^18^ and qualitative studies of individual workplace^38^ and national school^39^ PA policies. The RE-AIM framework has been operationalised to evaluate the impact of multisector partnerships to promote PA,^40^ but to our knowledge, this study is unique in its application to PA policy evaluation and provides a framework for understanding PA policy delivery. We suggest the approach could be replicated internationally to address the policy implementation gap highlighted by the WHO.^17^

Participants in our study suggested a need for guidance to ensure that PARS successfully targeted health inequalities. The standards highlight this as an area for attention, without giving specific guidance. Strategies that facilitate a reduction of health inequalities must be intersectoral and multidisciplinary in nature.^41^ As such, PARS must act as part of a wider system to provide greater investment in services to support deprived communities^42^ and currently underserved communities such as ethnic minority groups.^43^

### Limitations

This study included a large sample to ensure that the voices of different stakeholder groups were heard. We recognise that those who were aware and supportive of the standards may have been more likely to take part. This was especially true for those involved in the standards development. However, we mitigated against this by conducting internet searches to identify all PARS in Scotland and approached every scheme that we could find to invite them to participate. Two of the study authors were also involved in the standards development, and this may have influenced their interpretation of data. However, the lead analyst was not involved and was able to offer a more objective and independent perspective. Participants were not informed of author’s involvement in the standards, and the majority of interviews were not done by those involved. Some participants will have been aware, and this may have influenced their answers.

### Implications and future research questions

To enhance the delivery of the standards, our results suggest these needs: (1) a communication strategy to increase reach, especially for HCPs; (2) to address funding, resource, and capacity issues; (3) to address workforce recruitment, training and retention issues; (4) to explore and provide solutions to skills/capacity deficits related to the collection and interpretation of the proposed national dataset and (5) to explore how the credibility of PARS can be enhanced in health collaborations. We also suggest these recommendations could apply to future public health policy and guidance in Scotland and beyond and help to address the policy implementation gap.

Future research could explore longer-term adoption, implementation, effectiveness and maintenance of the standards. The role of the minimum dataset requires further exploration to balance sufficient information to encourage use versus maintaining utility for future research. If this can be managed, it may be possible to understand what works and what does not work (and why) when it comes to the huge range of options for PARS.

## Conclusion

Existing strong links between Scottish PARS stakeholders meant that awareness and likelihood of implementation of the standards were high. Delivery could be enhanced by a comprehensive communication strategy and addressing the lack of funding, workforce delivery capacity and the skills/capacity required to collect and interpret the proposed minimum national dataset.

## supplementary material

10.1136/bmjopen-2024-089723 online supplemental file 1

10.1136/bmjopen-2024-089723 online supplemental file 2

10.1136/bmjopen-2024-089723 online supplemental file 3

10.1136/bmjopen-2024-089723 online supplemental file 4

## Data Availability

Data are available upon reasonable request.
