# Supplementary material for: Evaluation of the initial rollout of the physical activity referral standards policy in Scotland: a qualitative study
Source: BMJ Open. 2025 Jan 23;15(1):e089723. doi: 10.1136/bmjopen-2024-089723 (PMC11758693; doi:10.1136/bmjopen-2024-089723)
Supplement: online supplemental file 1 [file bmjopen-15-1-s001.docx]

Supplementary File 1 Logic Model


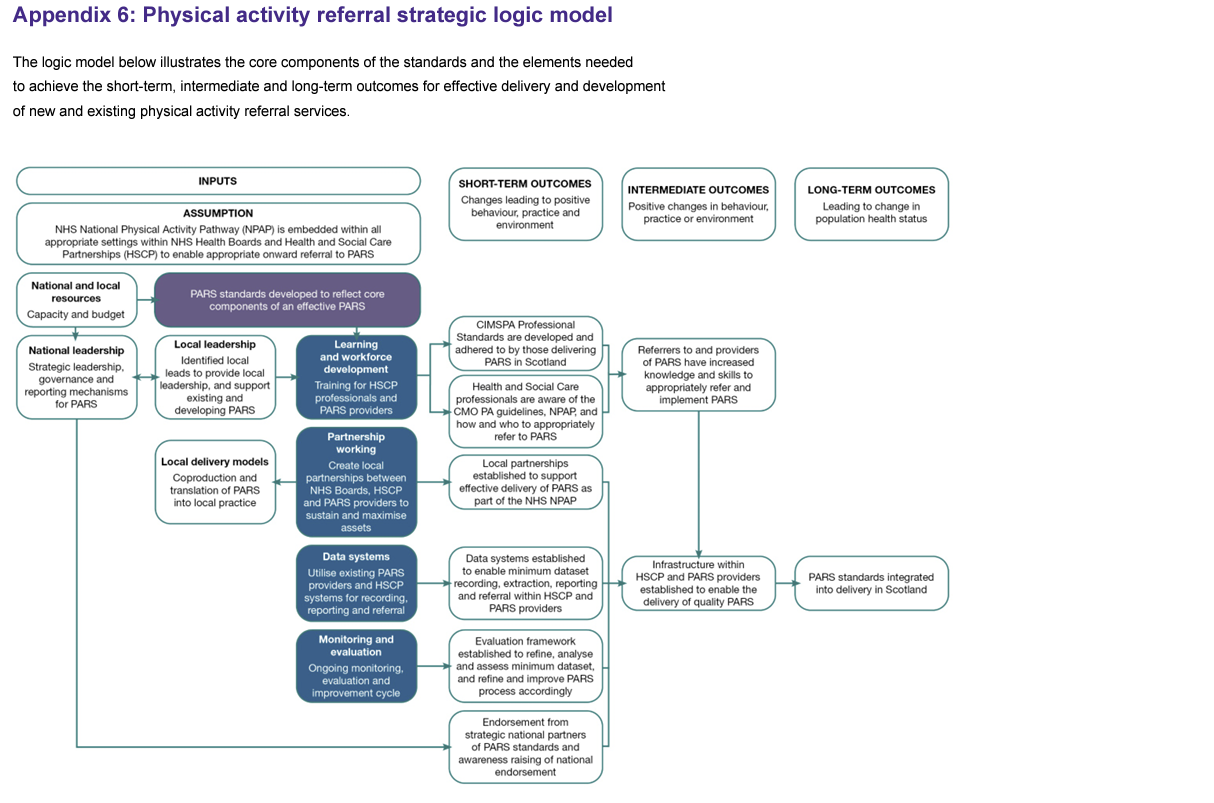


Source: Public Health Scotland. National Physical Activity Referral Standards. https://www.publichealthscotland.scot/publications/physical-activity-referral-standards/: Public Health Scotland,; 2022. Reproduced with permission from Public Health Scotland (granted 05/11/2024).
