# Supplementary material for: Evaluation of the initial rollout of the physical activity referral standards policy in Scotland: a qualitative study
Source: BMJ Open. 2025 Jan 23;15(1):e089723. doi: 10.1136/bmjopen-2024-089723 (PMC11758693; doi:10.1136/bmjopen-2024-089723)
Supplement: online supplemental file 2 [file bmjopen-15-1-s002.docx]

# Interview guides

Prior to interviews, researchers will introduce themselves and their interest and reasons for being involved in this study. Participants be reminded of the aim of the study and that that their answers will remain anonymous and direct quotes will only be used where identification can be avoided.

PARS senior managers

| **Question** | **Prompts** |
| --- | --- |
| Introductory questions | - *Can you start by telling me a bit about how long you have working in the leisure industry?* - *How big is the organisation that you are a senior manager in?* - *What area of Scotland do you work in? – is this predominately a rural or urban area?* |
| Please can you tell me a bit about your organisation’s involvement in physical activity referral schemes? | - *Just briefly, what sort of scheme does your organisation provide?* - *Where does your scheme fit in your corporate strategy?* - *How does your scheme fit with wider national and local strategic priorities?* - *What do you think the opportunities for your organisation are in the health/referral arena?* - *What are the risks to your scheme?* |
| Were you aware of the PHS PAR Standards prior to this study? | If no   - *In general, how useful do you think that the provision of a policy document focusing on Standards for PARS is? Why?* - *If useful: What needs to be done to raise awareness of the publication of the standards?* - *If not useful: What policy would be useful to help develop better PARS in the future?*   *If yes (dependent on answers to each):*   - *How did you find out about the Standards?* - *How aware of the detail of the standards are you?* - *What do you think is the most important aspect of the Standards?* - *How useful do you think that the provision of a policy document focusing on Standards for PARS is?* - *If useful: What needs to be done to raise awareness of the publication of the standards?* - *If not useful: What policy would be useful to help develop better PARS in the future?* |
| The standards propose a tiered approach to physical activity interventions on page 12. What do you think about this? | (Show, if possible, if not describe).   - *Were you aware of these tiers prior to this study?* - *How useful do you think the tiers are in the development of PA interventions like PARS?* - *What is needed to clarify the tiers?* - *How will your organisation implement the idea of tiers within the services you offer? If not, why not?* |
| The Standards contain six recommendations, and I would like to take a little time to find out your views about each recommendation. You do not need to know what the recommendations are to comment on this. | |
| **Partnership working**  Standard statement: Partnerships established between the health sector and physical activity referral services to enhance the development, delivery, and sustainability of quality physical activity referral services | - *How important do you think that partnership working is for your PARS?* - *Tell me about your existing PA partnerships with health and social care and other PA organisations* - *How do you engage with local partners and communities to make sure that the service meets the needs of the local population?* - *How do you link with other health related services such as weight management?* - *What, if anything, is the value of the Standards in helping to improve partnership working?* - *What, if anything, will you do differently as a result of the Standards publication?* |
| **Local delivery models**  No standard statement but local delivery models for should target inactive people with, or at risk of, one or more controlled long-term condition. Specific recommendations about aspects of delivery approach within Standards. | - *How important is developing a local delivery model in providing your PARS?* - *How does your organisation support those facing the greatest inequalities to access the scheme?* - *What are the biggest challenges in delivering a PARS to residents in your area?* - *What, if anything, is the value of the Standards in helping your organisation to focus your PARS delivery model? (Prompt with specific recommendations from the Standards if required).* - *What might your organisation do differently in light of the publication the Standards in terms of delivery model?* |
| **Learning and workforce development**  Standard statement: The workforce is equipped with the appropriate knowledge, skills and behaviours to design, deliver and evaluate effective physical activity referral services | - *The Standards advocate CIMSPA approved qualifications. What qualifications do your staff need to work for your PARS?* - *What, if any, behaviour change training do your staff complete?* - *What systems do you have in place for CPD?* - *How is PARS training paid for?* - *How might the Standards help in ensuring an appropriately trained workforce?* |
| **Data systems**  Standard statement: Data collection systems and procedures are streamlined and a minimum core dataset for physical activity referral established. | - *How important is a national minimum dataset as suggested in the Standards? Why?* - *What, if any, of the minimum national data set are you collecting? (Prompt as necessary)* - *What data systems does your organisation have in place to report activity for your PARS?* - *What do you do with the information that you currently collect?* - *What (if anything) needs to happen at a national level to ensure that the minimum national data set is implemented and useful?* - *What, if anything, will you do differently in light of the publication of the Standards?* |
| **Monitoring and evaluation**  Standard statement: Physical activity referral providers routinely monitor and evaluate their services as part of a continuous improvement cycle to enhance delivery. | - *What skills do you have within your organisation that allow for you to evaluate your PARS and develop improvement plans?* - *How does your organisation currently map PARS service delivery?* - *How useful is the PARS taxonomy contained in the Standards in potentially helping your organisation to map service delivery?* - *What would help your organisation to improve monitoring and evaluation for your PARS?* - *What, if anything will you do differently in light of the publication of the Standards?* |
| **Sharing learning and good practice**  Standard statement: Learning and good practice is routinely shared with and between service providers, practitioners, commissioners, and academia to enhance service delivery and the evidence base for physical activity referral. | - *How important is sharing learning and good practice for PARS?* - *How does your organisation currently share good practice about your PARS?* - *What is needed to facilitate better sharing of good practice for PARS as suggested by the Standards?* |
| What, if anything, will encourage your organisation to implement the Standards? | - *What needs to be done at a national level to encourage implementation of the Standards?* - *What needs to be done at organisational level to encourage implementation of the Standards?* |
| What are the barriers to your organisation implementing the Standards? | - *What could be done at national level to remove the barriers you have identified?* - *What could be done at organisational level to remove the barriers you have identified?* |
| Is there anything else you would like to tell me about the PHS PARS standards? | |

PARS co-ordinators

| **Question** | **Prompts** |
| --- | --- |
| Introductory questions | - *Can you start by telling me a bit about how long you have working in the leisure industry?* - *How big is the organisation that you are work in?* - *What area of Scotland do you work in? – is this predominately a rural or urban area?* |
| Please can you tell me a bit about your physical activity referral scheme? | - *Who can refer to the scheme?* - *Who can be referred to the scheme?* - *How long is the scheme?* - *What happens when someone is referred to the Scheme?* - *What activities are offered?* - *What type of locations are the activities offered in?* - *How much does it cost to attend the scheme?* - *What happens when people finish the scheme?* - *Approximately how many referrals do you get a year?* |
| Were you aware of the PHS PAR Standards prior to this study? | If no   - *How useful do you think that the provision of a policy document focusing on Standards for PARS is? Why?* - *What needs to be done to raise awareness of the publication of the standards?*   *If yes (dependent on answers to each):*   - *How did you find out about the Standards?* - *How useful do you think that the provision of a policy document focusing on Standards for PARS is? Why?* - *How aware of the detail of the standards are you?* - *What do you think is the most important aspect of the Standards?* - *How do you intend to use the Standards?* |
| The standards propose a tiered approach to physical activity interventions on page 12. What do you think about this? | (Show, if possible, if not describe).   - *How useful do you think the tiers are?* - *What is needed to clarify the tiers?* - *Which tiers does your organisation offer?* |
| **Partnership working**  Standard statement: Partnerships established between the health sector and physical activity referral services to enhance the development, delivery, and sustainability of quality physical activity referral services | - *How important is partnership working in achieving a high-quality PARS?* - *Tell me about your existing PA partnerships with health and social care (including any funding agreements)* - *How do you engage with local partners and communities to make sure that the service meets the needs of the local population?* - *How do you link with other health related services such as weight management?* - *What, if anything, is the value of the Standards in helping to improve partnership working?* - *What, if anything, will you do differently as a result of the Standards publication* |
| **Local delivery models**  No standard statement but local delivery models for should target inactive people with, or at risk of, one or more controlled long-term condition. Specific recommendations about aspects of delivery approach within Standards | - *How important is developing local delivery models in providing a high-quality PARS?* - *How can the Standards help in developing appropriate local delivery models?* - *How do you support those facing the greatest inequalities to access the scheme?* - *What are the biggest challenges in delivering a PARS to residents in your area?* |
| **Learning and workforce development**  The workforce is equipped with the appropriate knowledge, skills and behaviours to design, deliver and evaluate effective physical activity referral services | - *Tell me about the staffing model for your PARS* - *How might the Standards help in ensuring an appropriately trained workforce?* - *What qualifications do your staff need to work for your PARS?* - *What, if any, behaviour change training do your staff complete?* - *What systems do you have in place for CPD?* - *How is PARS training paid for?* |
| **Data systems**  Data collection systems and procedures are streamlined and a minimum core dataset for physical activity referral established. | - *How important is a national minimum dataset as suggested in the Standards and why?* - *What, if any, of the minimum national data set are you collecting? (Prompt as necessary)?* - *What data systems does your organisation have in place to report activity for your PARS?* - *What do you do with the information that you currently collect?* - *What needs to happen at a national level to ensure that the minimum national data set is implemented and useful?* |
| **Monitoring and evaluation**  Physical activity referral providers routinely monitor and evaluate their services as part of a continuous improvement cycle to enhance delivery. | - *What skills do you have within your organisation that allow for you to evaluate your PARS and develop improvement plans?* - *How does your organisation currently map PARS service delivery?* - *How can the PARS taxonomy contained in the Standards help your organisation to map service delivery?* - *What training does your organisation need to help with monitoring and evaluation?* |
| **Sharing learning and good practice**  Learning and good practice is routinely shared with and between service providers, practitioners, commissioners, and academia to enhance service delivery and the evidence base for physical activity referral. | - *How important is sharing learning and good practice in providing a high quality PARS?* - *How does your organisation currently share good practice about your PARS?* - *What is needed to facilitate better sharing of good practice for PARS as suggested in the Standards?* |
| What will encourage your organisation to implement the Standards? | - *What needs to be done at a national level to encourage implementation of the Standards?* - *What needs to be done at organisational level to encourage implementation of the Standards?* |
| What are the barriers to your organisation implementing the Standards? | - *What could be done at national level to remove the barriers you have identified?* - *What could be done at organisational level to remove the barriers you have identified?* |
| Is there anything else you would like to tell me about the PHS PARS standards? | |

Health and social care professionals

| **Question** | **Prompts** |
| --- | --- |
| Introductory questions | - *Can you start by telling me a bit about how long you have worked as a health or social care professional?* - *How big is the organisation (e.g., surgery) that you are work in?* - *What area of Scotland do you work in? – is this predominately a rural or urban area?* |
| Please can you tell me a bit about the physical activity referral scheme that you can refer to? | - *What organisation(s) can you refer patients to?* - *What do you know about what happens at the scheme?* - *What type of patients would you refer (if any)? (If not, why not?)* - *How do you decide who to refer?* - *How your decision about who to refer influenced by the National Physical Activity Pathway?* - *How do you know how your patients get on?* |
| Were you aware of the PHS PAR Standards prior to this study? | If no   - *How useful do you think that the provision of a policy document focusing on Standards for PARS is? Why?* - *What needs to be done to raise awareness of the publication of the standards?*   *If yes (dependent on answers to each):*   - *How did you find out about the Standards?* - *How useful do you think that the provision of a policy document focusing on Standards for PARS is? Why?* - *How aware of the detail of the standards are you?* - *What do you think is the most important aspect of the Standards?* - *How do you intend to use the Standards?* |
| The standards propose a tiered approach to physical activity interventions on page 12. What do you think about this? | (Show, if possible, if not describe).   - *How useful do you think the tiers are in helping to direct your patients towards appropriate PA options?* - *What is needed to clarify the tiers?* |
| **Partnership working**  Partnerships established between the health sector and physical activity referral services to enhance the development, delivery, and sustainability of quality physical activity referral services | - *Tell me what you know about any existing health and social care partnerships with organisations delivering PA interventions* - *What do you think could be done differently to improve these partnerships?* - *How might the publication of the Standards help to establish stronger partnerships?* |
| **Local delivery models**  No standard statement but local delivery models for should target inactive people with, or at risk of, one or more controlled long-term condition | - *How important is a localised approach for the PARS you can refer to?* - *What are the biggest challenges in referring your patients to your local PARS?* - *What might the PARS do differently to help address local health inequalities?* - *How can the Standards help in developing appropriate local delivery models?* |
| **Learning and workforce development**  The workforce is equipped with the appropriate knowledge, skills and behaviours to design, deliver and evaluate effective physical activity referral services | - *What do you know about the qualifications required for staff working for a PARS?* - *How might the Standards help in ensuring an appropriately trained workforce?* |
| **Data systems**  Data collection systems and procedures are streamlined and a minimum core dataset for physical activity referral established. | - *How important is a national minimum dataset as suggested in the Standards and why?* - *What needs to happen at a national level to ensure that the minimum national data set is implemented and useful?* |
| **Monitoring and evaluation**  Physical activity referral providers routinely monitor and evaluate their services as part of a continuous improvement cycle to enhance delivery. | - *How important is it for you to know about the performance of PARS that you are able to refer to?* - *How would you expect to be informed about performance?* - *The Standards contain a taxonomy for Providers to map service delivery. What do you think about this idea?* |
| **Sharing learning and good practice**  Learning and good practice is routinely shared with and between service providers, practitioners, commissioners, and academia to enhance service delivery and the evidence base for physical activity referral. | - *How does your surgery/organisation currently hear about PARS good practice?* - *What is needed to facilitate better sharing of good practice for PARS as suggested in the Standards?* |
| What will encourage your organisation to implement the Standards? | - *What needs to be done at a national level to encourage implementation of the Standards?* - *What needs to be done within your organisation to encourage implementation of the Standards?* |
| What are the barriers to your organisation implementing the Standards? | - *What could be done at national level to remove the barriers you have identified?* - *What could be done at organisational level to remove the barriers you have identified?* |
| Is there anything else you would like to tell me about the PHS PARS standards? | |

Policy development/implementation professionals

| **Question** | **Prompts** |
| --- | --- |
| Introductory questions | - *Can you start by telling me a bit about how long you have worked in policy development or implementation?* - *How big is the organisation that you are work in?* - *What area of Scotland do you work in? – is this predominately a rural or urban area?* |
| Please can you tell me what you know about physical activity referral schemes? | - *If limited awareness, give a short explanation of PARS* - *How important do you think that PARS are in increasing PA in Scotland?* - *How do they fit with wider PA policy in Scotland?* |
| Were you aware of the PHS PAR Standards prior to this study? | If no   - *How useful do you think that the provision of a policy document focusing on Standards for PARS is? Why?* - *What needs to be done to raise awareness of the publication of the standards?*   *If yes (dependent on answers to each):*   - *How did you find out about the Standards?* - *How useful do you think that the provision of a policy document focusing on Standards for PARS is?* - *How aware of the detail of the standards are you?* - *What do you think is the most important aspect of the Standards?* - *From a policy implementation perspective, who should be using the Standards and how?* |
| The standards propose a tiered approach to physical activity interventions on page 12. What do you think about this? | (Show, if possible, if not describe).   - *How useful do you think the tiers are in influencing how healthcare professionals and PA providers promote and support PA?* - *What is needed to clarify the tiers?* |
| **Partnership working**  Partnerships established between the health sector and physical activity referral services to enhance the development, delivery, and sustainability of quality physical activity referral services | - *How might the publication of the Standards help to establish stronger partnerships between the health sector and organisations delivering PA interventions?* - *What do you think could be done differently to improve these partnerships?* - *How can partnerships between leisure trust and third sector organisations be strengthened to promote a wide range of PA options within referral structures (If appropriate to interviewee)?* |
| **Local delivery models**  No standard statement but local delivery models for should target inactive people with, or at risk of, one or more controlled long-term condition | - *How important is a localised approach for PA interventions like PARS?* - *How can the Standards help in developing appropriate local delivery models?* - *What might the PARS do differently to help address local health inequalities?* |
| **Learning and workforce development**  The workforce is equipped with the appropriate knowledge, skills and behaviours to design, deliver and evaluate effective physical activity referral services | - *How might the Standards help in ensuring an appropriately trained workforce?* |
| **Data systems**  Data collection systems and procedures are streamlined and a minimum core dataset for physical activity referral established. | - *How important is a national minimum dataset as suggested in the Standards and why?* - *What needs to happen at a national level to ensure that the minimum national data set is implemented and useful?* |
| **Monitoring and evaluation**  Physical activity referral providers routinely monitor and evaluate their services as part of a continuous improvement cycle to enhance delivery. | - *How important is robust internal monitoring and evaluation for PA interventions like PARS?* - *How does this differ between organisations like leisure trusts and third sector providers?* - *The Standards contain a taxonomy for Providers to map service delivery. What do you think about this idea?* |
| **Sharing learning and good practice**  Learning and good practice is routinely shared with and between service providers, practitioners, commissioners, and academia to enhance service delivery and the evidence base for physical activity referral. | - *How does your surgery/organisation currently hear about PARS good practice?* - *What is needed to facilitate better sharing of good practice for PARS as suggested in the Standards?* |
| What will encourage your organisation to implement the Standards? | - *What needs to be done at a national level to encourage implementation of the Standards?* - *What needs to be done within your organisation to encourage implementation of the Standards?* |
| What are the barriers to your organisation implementing the Standards? | - *What could be done at national level to remove the barriers you have identified?* - *What could be done at organisational level to remove the barriers you have identified?* |
| Is there anything else you would like to tell me about the PHS PARS standards? | |
