# Supplementary material for: Evaluation of the initial rollout of the physical activity referral standards policy in Scotland: a qualitative study
Source: BMJ Open. 2025 Jan 23;15(1):e089723. doi: 10.1136/bmjopen-2024-089723 (PMC11758693; doi:10.1136/bmjopen-2024-089723)
Supplement: online supplemental file 3 [file bmjopen-15-1-s003.docx]

Supplementary File 3: Themes
