# Supplementary figures and images for: Evaluation of the initial rollout of the physical activity referral standards policy in Scotland: a qualitative study
Source: BMJ Open. 2025 Jan 23;15(1):e089723. doi: 10.1136/bmjopen-2024-089723 (PMC11758693; doi:10.1136/bmjopen-2024-089723)

Supplementary file 4: Excerpt from NVIVO matrix


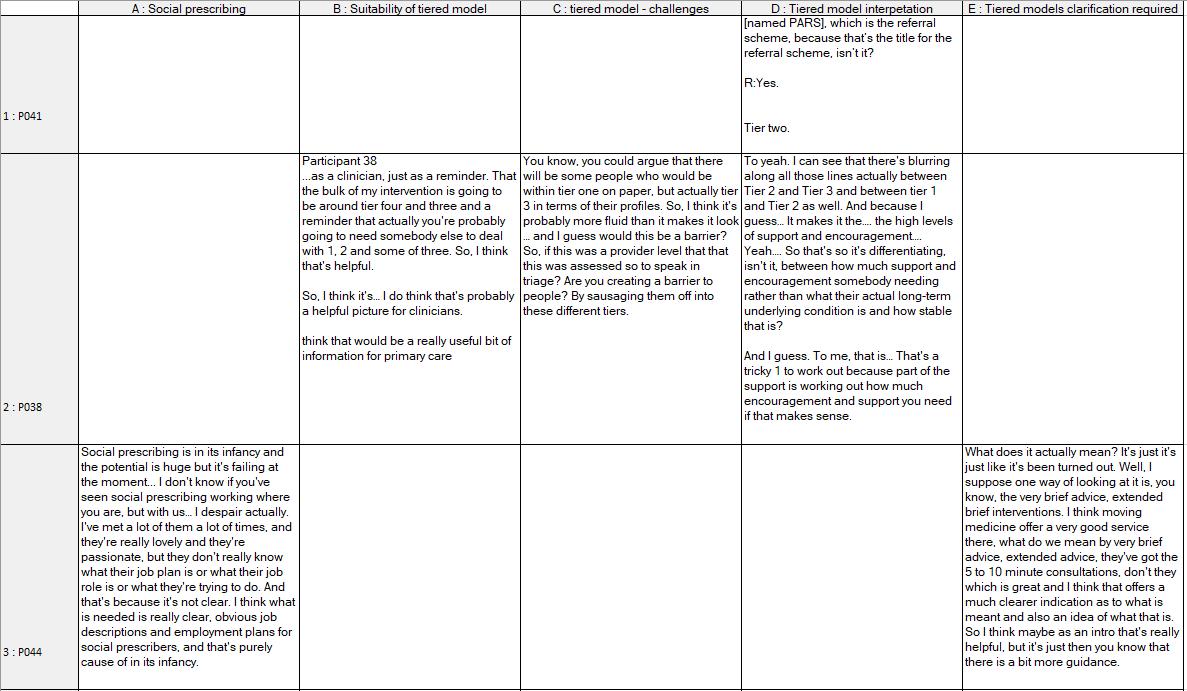


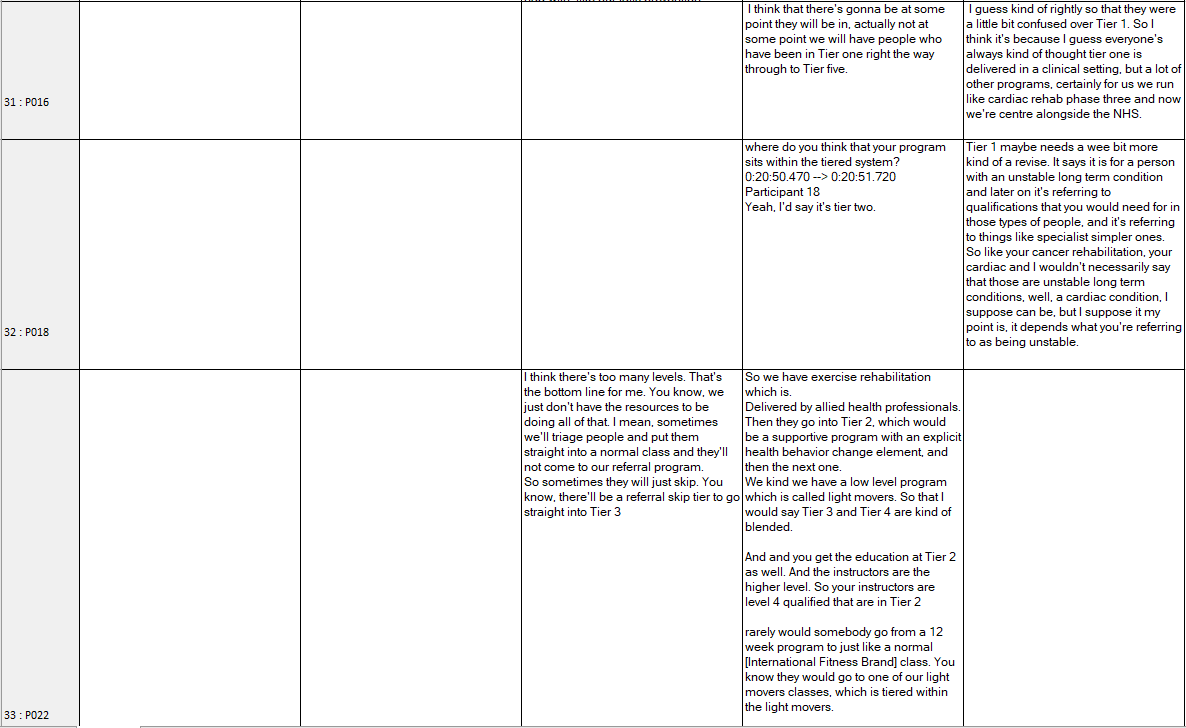


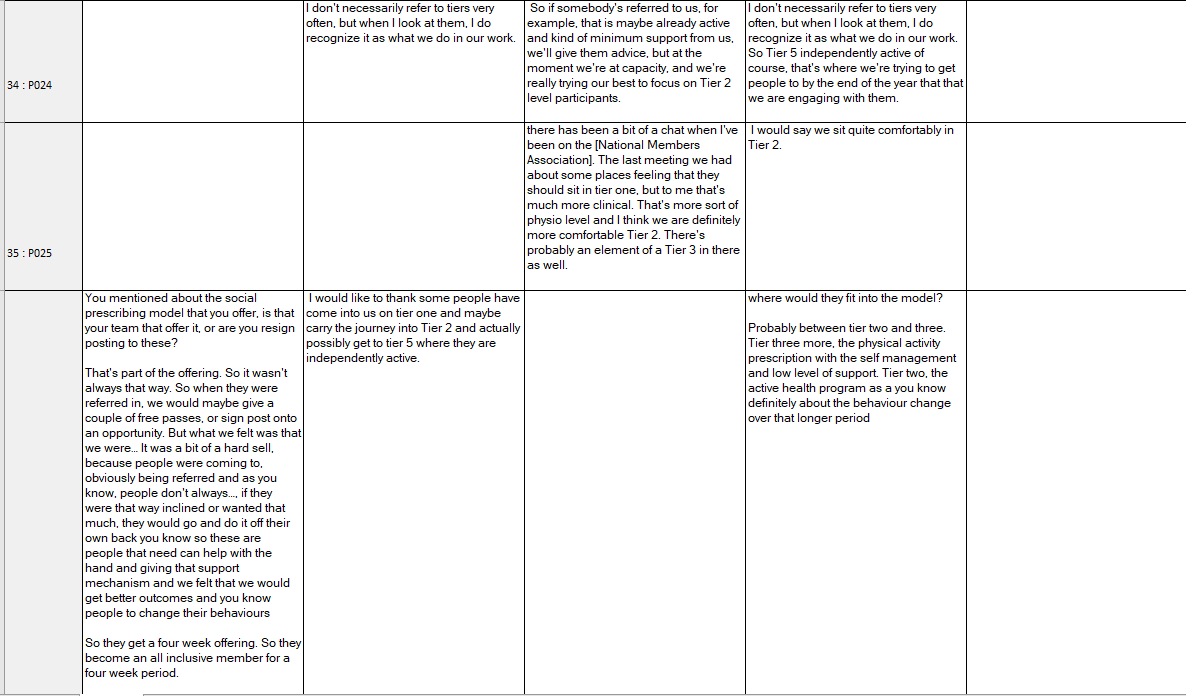

Supplement: online supplemental file 4 [file bmjopen-15-1-s004.docx]
