# Supplementary material for: Evaluation of the initial rollout of the physical activity referral standards policy in Scotland: a qualitative study
Source: BMJ Open. 2025 Jan 23;15(1):e089723. doi: 10.1136/bmjopen-2024-089723 (PMC11758693; doi:10.1136/bmjopen-2024-089723)
Supplement: Supplementary file 5 [file bmjopen-2024-089723.reviewer_comments.pdf]

PEER REVIEW HISTORY

BMJ Open publishes all reviews undertaken for accepted manuscripts. Reviewers are asked to complete a checklist review form and are provided with free text boxes to elaborate on their assessment. These free text comments are reproduced below.

ARTICLE DETAILS

Title (Provisional)

An evaluation of the initial rollout of the Physical Activity Referral Standards policy in Scotland: A qualitative study

Authors

Hanson, Coral L; Mchale, Sheona; Neubeck, Lis; Dougall, Nadine; Kelly, P

VERSION 1 - REVIEW

**Reviewer**	**1**
**Name**	**Tremblett, Madeleine**
**Affiliation**	**University of the West of England**
**Date**	**24-Jul-2024**
**COI**	**none**

Overall this was a really interesting and well written paper, but throughout I think a bit more clarity on the methods/approach would be helpful.

E.g.In the objectives (abstract) you state the aim was to evaluate the reach, adoption and implementation of the programme, and then these became the themes. With thematic analysis this is quite problematic and shows a low level of intepretation of the interview data. I think perhaps it might have been clearer to frame this as a framework analysis, if this would eventually show that interpretation was constrained in advance by this framework.

I think it would be helpful to clarify for the reader the type of epistemological approach this study followed, particularly to help frame the analytic findings.

In the findings, I was unclear on some of the themes, which felt more like categories from a thematic analysis approach (but perhaps this is not really the apprpoach - see https://www.tandfonline.com/doi/full/10.1080/14780887.2020.1769238) - I found the use of the term effectiveness confusing, when it really all relates to impact - so my thought was why not call impact?

Throughout the results I found there was limited comment on the underlying critique of the PARS system, that seems present in almost all of the quotes used to support - - e.g. which

seems to give off the idea that it is a nice idea but very difficult to implement in practice. I wonder how this could be bought into the paper to reflect ideas present from participants.

Again, from a thematic analytic perspective there seems high level of overlap between implementation/adoption.

Often there are some jargonistic terms used, which perhaps some more plain english would make clearer - e.eg reference to 'the minimum dataset'.

The main conclusion in the discussion is that there is success in this programme, but again, this seems to bury some of the negative issues highlighted by interviewees.

There is missing some suggestion of what the limitations of the study are.

I have suggested major revision, but I think this would hinge on how you want to align the methodological approach to the results of the study.

**Reviewer**	**2**
**Name**	**Hewison, Ann**
**Affiliation**	**University of York, Department of Health Sciences**
**Date**	**09-Sep-2024**
**COI**	**I have no potentially competing or conflicting interest to**
**declare**	

Thank you for the opportunity to review this interesting paper. The study demonstrates the value of using a qualitative design to determine the complexity of how successfully policy is adopted and implemented, and where gaps and challenges exist. It is written clearly, and the tables/figures well designed. It makes conclusions which appear relevant to the area of practice. I have suggested changes which I would consider minor, and I understand that word limit may have had an influence in these areas:

Study objectives/Abstract

• The abstract states: “This study evaluated initial reach, adoption, and implementation of the Standards.” I think ‘effectiveness’ needs adding here as this was a category/theme in the findings?

• The study findings were all from the perspectives of stakeholders, making this clear in the abstract objectives would help the reader understand the study.

• The abstract states that thematic analysis was used, however the methods section seems to describe a Framework approach: see my later comment under Methods.

Methods

• It would be useful to know what key characteristics the purposive sampling was based on.

• A statement about what participant information was provided and the consent process/confidentiality arrangements will help justify the ethics of the study.

• A brief description of the stakeholder roles would be useful for those not familiar with them, for example what do PARS coordinators do?

• Using a purely qualitative design provided comprehensive findings but it would be interesting to know why this was chosen, rather than for e.g. a mixed methods design.

• The use of the Framework approach and RE-AIM categories seem very appropriate, and the stages of a Framework analysis are described, but I am not sure how thematic analysis was also used as an approach? I understand there are similarities in these approaches, but further explanation would be helpful. Was this use of thematic analysis referring to the identification of a thematic/analytical framework, which is a stage in the Framework approach?

• It would be interesting to understand why the RE-AIM framework was chosen to support the analysis.

• It is interesting that the team decided to include the RE-AIM theme/category 'Effectiveness' given the qualitative study design and the definition of effectiveness, later in the results, as "what impact the Standards had on PARS outcomes". An explanation of how the qualitative approach was intended to measure this may strengthen the methodology as, for example, some readers may expect effectiveness to be measured using a trial design.

• Reflexivity: Two of the analysts were involved in the standards development: how you feel this effected your outlook?

• A statement about measures taken to enhance the rigour of the study may strengthen this section for e.g. triangulation using multiple analysts to support study credibility/validity.

Results

• A statement reflecting on whether you think you caught the key characteristics, and a good spread of participants would support its transferability here.

• Effectiveness: see previous comments. The definition is interesting given that the results suggested that there was not enough evidence to do this due to the early stage of the Standards rollout. Some may also argue that measuring effectiveness requires a mixed methods or quantitative approach. There are valuable findings, however, on how participants were planning to map and measure the impact of the standards. You may wish to re-consider the definition of effectiveness, or add some reflection on this in the discussion?

Discussion/conclusion

• The Principal findings state “We analysed stakeholder views...considering the reach, adoption, and implementation elements of the RE-AIM framework”, should ‘Effectiveness’ be included as this was a category/theme in the findings?

• In the Principal findings it would be useful to emphasise the difficulty assessing effectiveness due to the early stage of the Standards, also see earlier comment on Effectiveness in the Results section.

• The assessment of inequalities and provision of PARS to marginalised groups seemed to be a common theme in the ‘Effectiveness’ Results section, participants wanting more guidance on addressing equality issues. Including this in the discussion would seem important.

• I am not sure where Implication number 5 “to explore how credibility of PARS can be enhanced in health collaborations” is supported in the findings, this may need a little explanation.

References

• The reference for Framework approach appears to be about a tool to assess the quality of qualitative evaluations, not the Framework approach process, such as the commonly cited: Gale, NK et al (2013): http://www.biomedcentral.com/1471-2288/13/117

Reviewer **3**

Name **Kesten, Joanna**

Affiliation **University of Bristol, NIHR CLAHRC West and NIHR Health Protection Research Unit in Evaluation of Interventions**

Date **17-Sep-2024**

COI **None to report.**

Thank you for the opportunity to review this interesting manuscript seeking to evaluate the initial rollout of the Physical Activity Referral Standards policy in Scotland. Overall, I think the reporting is comprehensive and clear. I have highlighted a few areas which I believe could be more transparently reported, particularly in relation to the sample’s involvement and familiarity with the Standards, the analysis approach and consideration of the study’s methodological strengths and limitations.

Abstract

In the abstract and main text please provide more detail on healthcare professionals involved in PARS (e.g. primary care, secondary care, GPs, nurses).

Setting: you could include details about whether the sample included stakeholders from urban and rural areas.

Participants: You could state the numbers from each stakeholder group (scheme providers, healthcare professionals, policy stakeholders) in the abstract to give an idea of the spread of the data.

Findings: A brief explanation of what a minimum dataset is for an unfamiliar audience would be beneficial.

Strengths and limitations of this study

Please also consider whether the views captured may reflect more positive perceptions of the Standards and greater awareness of them than among those who did not participate? What did the team do to mitigate against this (see comment on this below too)?

Introduction

The introduction is clear and comprehensive.

I wonder if it would also be helpful to expand a little on the mechanisms through which PARS are expected to work, potentially referring to or presenting a logic model?

In addition, I note that the National Centre for Sport and Exercise Medicine has worked with partners to produce a toolkit for the design, implementation and evaluation of exercise and referral schemes. Would it be helpful to cite this toolkit and how it sits alongside the Public Health Scotland Standards? https://ncsem-em.org.uk/wp-content/uploads/2020/10/section-8-evaluating-exercise-referral-schemes.pdf

The final paragraph touches on the translational gap between evidence and practice. To expand on this and strengthen the rationale for this research, I wonder whether it would be useful to draw on literature around knowledge mobilisation, implementation science or theories such as Normalization Process Theory. RE-AIM is used in the analysis, describing this framework (and/or other literature) in the introduction could help explain the importance of understanding implementation processes to enhance delivery and uptake.

Study design

Could the authors add a little information on the selection of qualitative methods (as opposed to a mixed methods approach) to address the aim of this research? Is this included in the wider project which is referred to?

PPI

Please can you clarify what is meant by a PARS participant – are these patients who have been referred to PAR?

It would be great to know a little more about the ways in which this involvement shaped the study methods and findings.

Sample

Please expand on the description of the purposive sampling approach. Which stakeholder characteristics/roles or experiences were you seeking?

Similarly, what were the inclusion / exclusion criteria for the study? Were any of the interviewees involved in the development or roll out of the Standards, for example?

This is hinted at in the data collection and discussion sections but could be more transparently reported and reflected on in a strengths and limitations section (see comments below).

For qualitative research you interviewed a large sample. Please include an explanation about how you decided when to stop data collection.

Data collection

There is a good level of detail in this section.

It could also be worth clarifying (in line with the topic guides) what information was provided to the participants about the Standards / PARS particularly if their responses indicated they were not (very) aware of them?

Analysis

Both thematic analysis and framework analysis are employed. Can the authors provide a rationale for the inclusion of both approaches please?

My primary concern about the analysis and reporting is that reclassifying the inductive themes into RE-AIM means they not apparent in the reporting. Could the authors consider presenting inductive themes as subthemes or more clearly illustrating how the initial inductive themes fit within RE-AIM?

The results state: "Enablers and barriers for the Standards rollout were mapped within the reach, effectiveness, adoption, and implementation elements of RE-AIM." I wonder

if this sentence fits better in the data collection or analysis section which do not mention enablers and barrier questions/themes/codes?

The analysis also does not mention the quantification of the findings presented in the results section.

Results

The findings are mostly clearly reported and are valuable in understanding the early implementation of the Standards.

Please clarify, how was 'awareness of the standards' defined as 'high'? Does this reflect involvement in developing the standards (see earlier comment)? This is noted in the discussion but could be reported in the findings as an explanation, particularly if it is evident in the data.

Please explain what tiered models of PA interventions means for unfamiliar audience.

As above, please expand on what a ‘minimum national data set’ is for an unfamiliar audience.

Discussion

Implications

The implications of this work are valuable for supporting the adoption of the Standards.

Could the authors describe if any work has been done (or is planned) to mobilise this evidence and enhance delivery of the standards in partnership with key stakeholder groups please?

Strengths and limitations

Please add a ‘strengths and limitations’ section to the discussion section. As above, one point to consider is whether there is a potential bias in the sample towards those who are aware and supportive of the standards? How likely is it that people who did not know about the standards at all would take part? What did you do to mitigate against this? In addition, please reflect on the possible impacts (including beneficial) of the study authors involvement in the development of the Standards (e.g. including the interpretation of the data). Did participants know about the study authors involvement in the development of the Standards? If so, what efforts were made to mitigate against social desirability bias?

Comparison to previous literature – it may also be useful to situate your findings in the context of the systems based literature. E.g. https://bmcmedresmethodol.biomedcentral.com/articles/10.1186/s12874-022-01570-4

VERSION 1 - AUTHOR RESPONSE

Reviewer: 1<br>Overall this was a really interesting and well written paper, but throughout I think a bit more clarity on the methods/approach would be helpful.	Thank you for your feedback. In the sections below we have included additional details regarding the methods and approach we used.
In the objectives (abstract) you state the aim was to evaluate the reach, adoption and implementation of the programme, and then these became the themes. With thematic analysis this is quite problematic and shows a low level of interpretation of the interview data. I think perhaps it might have been clearer to frame this as a framework analysis, if this would eventually show that interpretation was constrained in advance by this framework.	Thank you for this comment, which is reflected by the other reviewers. We have amended the abstract to read:<br><br>We analysed data using the framework

	method within the context of the RE-AIM framework.<br><br>We have also amended relevant sections in the method (Please see later comments)
I think it would be helpful to clarify for the reader the type of epistemological approach this study followed, particularly to help frame the analytic findings.<br><br>I have suggested major revision, but I think this would hinge on how you want to align the methodological approach to the results of the study.	Thank you for suggesting this, we have amended the initial sentence in the study design section to read:<br><br>This study was an observational evaluation, situated within a constructivist, interpretative epistemological approach, of a national policy roll out using qualitative methods.<br><br>We have addressed all reviewers comments to make the methodological approach clear.
In the findings, I was unclear on some of the themes, which felt more like categories from a thematic analysis approach (but perhaps this is not really the approach - see https://www.tandfonline.com/doi/full/10.1080/14780887.2020.1769238)	To address reviewers' concerns, we have updated our data analysis section in the manuscript to clarify our methodological approach as using the Framework method. We have added additional detail to the data analysis section and included a supplementary file offering one example of the NVivo

	framework matrices used during the charting phase:<br><br>We have amended the Methods section to read:<br>“Interviews were transcribed verbatim, anonymised, and imported into NVivo20, a data analysis organisation tool. Data were analysed using the Framework Method (20). During the familiarisation phase, SM and CLH checked transcripts with audio recordings for accuracy. They independently created inductive open codes (n=97) for three transcripts in each stakeholder group. In the identification phase they grouped open codes into 12 initial inductive themes (awareness, usefulness, the tiered model of PA interventions, local delivery contexts, cost, inequalities, sharing good practice, workforce issues, data systems, monitoring/evaluation, communication, and partnerships). Initial themes were mapped to the reach, adoption, and implementation

	elements of the RE-AIM framework (21, 22) to structure the findings. This initial framework was applied as a tool for interpretation, as opposed to theme development, and presented to the other researchers during a data workshop. After a critical discussion, the inductive themes were reorganised within the RE-AIM framework to include effectiveness. During indexing, SM coded 10 more transcripts, which CLH checked for coding accuracy by reading code content and using NVIVO20 deductive word searching. SM then coded all remaining transcripts. NVivo framework matrices were used during the charting phase, (example supplementary file X). Researchers explored and rearranged the data within the inductive themes and compared across cases to develop explanatory summaries. Prior to final analysis, the framework was reviewed, and the analysis mapped to

	the reach, effectiveness, adoption, and implementation elements of RE-AIM.”
Again, from a thematic analytic perspective there seems high level of overlap between implementation/adoption.	Thank you for this feedback. We have rephrased the definitions for adoption and implementation to provide more clarity and revisited our coding in light of this.<br>We defined adoption as understanding what elements of the Standards influence decisions about use and in what settings.<br><br>We defined implementation as understanding the barriers and facilitators to actioning the Standards.<br><br>We have moved utility of the minimum national data set to adoption and funding to implementation as we feel this better reflects the revised definitions. We have moved some text from the implementation to adoption as a result.<br><br>In adoption:<br>The most discussed element of the Standards was the utility of the

	proposed minimum national data set for future evaluation and the data systems that were required to collect this. While the minimum dataset was considered important, not all providers intended to collect all elements of it. For PARS providers, this was because it did not align to funders reporting requirements or because of a perceived lack of value of the measures. Additionally, some HCPs and PARS providers expressed apprehension about the extent of measures and raised questions about the benefits of collecting such data. Other HCPs questioned why adoption of the Standards was not mandatory across Scotland to standardise approaches.<br><br>In implementation: We did not need to change this as funding was already mentioned and so we have removed the duplication that was in adoption by this refinement.<br><br>We have also reviewed the quotes

	used for the two elements and have changed two quotes between themes and added two more:<br><br>In adoption:<br>“But the PARS ask for too much, that’s the one negative side. And I understand why they ask that, and in an ideal world it would be great to have all this information, but it's a huge amount of information.” (Participant 62) (added)<br><br>“I mean we don't use all of these things. I think our questionnaires are tailored very specifically to report back to our funders.” (Participant 64) (moved from implementation)<br><br>In implementation<br>“The only thing I would say about that referral guidelines, is it makes for an expensive program.” (Participant 22) (added)<br><br>“There's huge pressures on leisure facilities in terms of income, generations, staffing, etc… what we really need to do is… we need to bring in some more

	investment so we can actually give them the protected time they need to deliver the level of scheme that they want to deliver." (Participant 21) (moved from adoption)
Often there are some jargonistic terms used, which perhaps some more plain English would make clearer - e.eg reference to 'the minimum dataset'.<br><br>The main conclusion in the discussion is that there is success in this programme, but again, this seems to bury some of the negative issues highlighted by interviewees.<br><br>There is missing some suggestion of what the limitations of the study are.	We have clarified what the minimum data set is in the abstract:<br><br>Views about the minimum dataset (suggested essential or desirable data fields to be collected for monitoring and evaluation)<br><br>And have added in further detail at the end of the introduction to read: … a national minimum data set, which included suggested essential or desirable data fields to be collected for monitoring and evaluation such as demographics, referral numbers, uptake and adherence)<br><br>We have added in a Limitations section This study included a large sample to ensure that the voices of different Stakeholder groups were heard. We recognise that those who were aware and

	supportive of the Standards may have been more likely to take part. This was especially true for those involved in the Standards development. However, we mitigated against this by conducting internet searches to identify all PARS in Scotland and approached every scheme that we could find to invite them to participate. Two of the study authors were also involved in the Standards development and this may have influenced their interpretation of data. However, the lead analyst was not involved and was able to offer a more objective and independent perspective. Participants were not informed of author involvement in the Standards and the majority of interviews were not done by those involved. Some participants will have been aware, and this may have influenced answers.
Results:<br>I found the use of the term effectiveness confusing, when it really all relates to impact - so my thought was why not call impact?	Initially we did not include effectiveness in our analysis (as detailed in the analysis

Throughout the results I found there was limited comment on the underlying critique of the PARS system, that seems present in almost all of the quotes used to support - - e.g. which seems to give off the idea that it is a nice idea but very difficult to implement in practice. I wonder how this could be bought into the paper to reflect ideas present from participants.	section) but on discussion felt that some of the sub-themes did represent this element of the RE-AIM framework . This was because the sub-themes were focused on future outcomes.<br><br>We have redefined effectiveness in the manuscript as:<br><br>We defined effectiveness as what PARS outcomes result from actions taken as a consequence of the publication of the Standards.<br><br>Thank you for this comment. Much of the evidence and practice around PARS does indicate that it is to use your words, a very nice idea that is very difficult to implement in practice.<br><br>We have added the following sentence to the implementation section of the results:<br><br>All stakeholders were positive about the value of PARS, but responses reflected the challenges in

	implementing schemes.<br><br>However, as the paper is about implementation of the Standards, rather than implementation of PARS in general, we have not followed this through into the discussion, but have added into the new limitations section:<br><br>This study included a large sample to ensure that the voices of different Stakeholder groups were heard. We recognise that those who were aware and supportive of the Standards may have been more likely to take part. This was especially true for those involved in the Standards development. However, we mitigated against this by conducting internet searches to identify all PARS in Scotland and approached every scheme that we could find to invite them to participate. Two of the study authors were also involved in the Standards development and this may have influenced their interpretation of data. However, the

	lead analyst was not involved and was able to offer a more objective and independent perspective. Participants were not informed of author involvement in the Standards and the majority of interviews were not done by those involved. Some participants will have been aware, and this may have influenced answers.
Reviewer: 2<br>Dr. Ann Hewison, University of York<br>Comments to the Author:<br>Thank you for the opportunity to review this interesting paper. The study demonstrates the value of using a qualitative design to determine the complexity of how successfully policy is adopted and implemented, and where gaps and challenges exist. It is written clearly, and the tables/figures well designed. It makes conclusions which appear relevant to the area of practice. I have suggested changes which I would consider minor, and I understand that word limit may have had an influence in these areas:	Thank you for your positive feedback.
Study objectives/Abstract<br>• The abstract states: “This study evaluated initial reach, adoption, and implementation of the Standards.” I think ‘effectiveness’ needs adding here as this was a category/theme in the findings?<br>• The study findings were all from the perspectives of stakeholders, making this clear in the abstract objectives would help the reader understand the study.<br>• The abstract states that thematic analysis was used, however the methods section seems to describe a Framework approach: see my later comment under Methods.	Thank you for pointing out this omission, we have amended the abstract to address this and following point:<br><br>This study evaluated stakeholder perspectives of the initial reach, adoption, implementation and effectiveness of the Standards.<br><br>Thank you for your comment, we have

	amended the abstract to: We conducted thematic analysis via analysed data using the framework method within the context of the RE-AIM framework.
Methods<br><br>• It would be useful to know what key characteristics the purposive sampling was based on. A brief description of the stakeholder roles would be useful for those not familiar with them, for example what do PARS coordinators do?<br><br>• A statement about what participant information was provided and the consent process/confidentiality arrangements will help justify the ethics of the study.<br><br>• Using a purely qualitative design provided comprehensive findings but it would be interesting to know why this was chosen, rather than for e.g. a mixed methods design.<br><br>• The use of the Framework approach and RE-AIM categories seem very appropriate, and the stages of a Framework analysis are described, but I am not sure how thematic analysis was also used as an approach? I understand there are similarities in these approaches,	We thank you for your comment. We have added more detail in the section describing the stakeholder roles: A purposive sample of Scottish PARS stakeholders, including senior managers of PARS provider organisations (responsible for strategic planning for health and wellbeing activities), PARS coordinators (responsible for managing the day-to-day delivery of schemes), HCPs able to refer to PARS and those involved in developing and/or implementing PA policy (working in government, national sporting bodies, and Scottish NHS health boards).<br><br>We have added a section to the sample and researchers section. “Individuals were sent an online link in recruitment emails which provided

but further explanation would be helpful. Was this use of thematic analysis referring to the identification of a thematic/analytical framework, which is a stage in the Framework approach?<br><br>• It would be interesting to understand why the RE-AIM framework was chosen to support the analysis.<br><br>• It is interesting that the team decided to include the RE-AIM theme/category 'Effectiveness' given the qualitative study design and the definition of effectiveness, later in the results, as "what impact the Standards had on PARS outcomes". An explanation of how the qualitative approach was intended to measure this may strengthen the methodology as, for example, some readers may expect effectiveness to be measured using a trial design.<br><br>• Reflexivity: Two of the analysts were involved in the standards	access to a participant information sheet, privacy notice, and consent form. Participation was voluntary, and individuals gave online informed consent via the secure NOVI survey system (NOVI Survey v 8.8, 3rd Millennium, Cambridge, MA 02142, USA) to register for the study."<br><br>This study part of a larger funded project. The aim was to establish a baseline for aware of the Standards and intention to use. The part (currently underway) includes 1) a national survey of the design and delivery PARS, 2) collation of an anonymised dataset where PARS are collecting any aspects of the minimum national data set and 3) focus groups with participants to understand their views of how schemes should be monitored and evaluated. This means that overall it will be a mixed methods project, but we feel that this initial study is

development: how you feel this affected your outlook?<br>• A statement about measures taken to enhance the rigour of the study may strengthen this section for e.g. triangulation using multiple analysts to support study credibility/validity.	distinct and worthy of separate publication.<br>We have added in a shorter statement in the study design section to reflect the above:<br><br>This study was the first part of a larger project that includes a mixed methods approach to explore the design and delivery PARS, monitoring and evaluation strategies and participants views of how schemes should be monitored and evaluated.<br><br>This comment has been reflected by the other reviewers, so our apologies for the lack of clarity: We have amended the Methods section to read:<br><br>"Interviews were transcribed verbatim, anonymised, and imported into NVivo20, a data analysis organisation tool. Data were analysed using the Framework Method (20). During the familiarisation phase, SM and CLH checked transcripts with audio recordings for accuracy. They independently

	created inductive open codes (n=97) for three transcripts in each stakeholder group. In the identification phase they grouped open codes into 12 initial inductive themes (awareness, usefulness, the tiered model of PA interventions, local delivery contexts, cost, inequalities, sharing good practice, workforce issues, data systems, monitoring/evaluation, communication, and partnerships). Initial themes were mapped to the reach, adoption, and implementation elements of the RE-AIM framework (21, 22) to structure the findings. This initial framework was applied as a tool for interpretation, as opposed to theme development, and presented to the other researchers during a data workshop. After a critical discussion, the inductive themes were reorganised within the RE-AIM framework to include effectiveness. During indexing, SM coded 10 more transcripts, which CLH checked for

	coding accuracy by reading code content and using NVIVO20 deductive word searching. SM then coded all remaining transcripts. NVivo framework matrices were used during the charting phase, (example supplementary file X). Researchers explored and rearranged the data within the inductive themes and compared across cases to develop explanatory summaries. Prior to final analysis, the framework was reviewed, and the analysis mapped to the reach, effectiveness, adoption, and implementation elements of RE-AIM."<br><br>The RE-AIM framework was chosen as the theoretical framework for the whole project. When this study is combined with the other elements as described above, we considered that it would offer an excellent way of understanding the overall results.<br><br>Initially we did not include

	effectiveness in our analysis (as detailed in the analysis section) but on discussion felt that some of the sub-themes did represent this element of the RE-AIM framework . This was because the sub-themes were focused on future outcomes.<br><br>We have redefined effectiveness in the manuscript as:<br><br>We defined effectiveness as what PARS outcomes result from actions taken as a consequence of the publication of the Standards.<br>We have also clarified elsewhere that this is part of a larger study, where we will examine effectiveness more thoroughly. We felt it was important to highlight the elements included in this analysis as potentially influencing future effectiveness of PARS – rather than the implementation of the Standards.<br><br><br>Thank you for your comments, we added a statement into researchers

	section of the methods. Two authors (PK and CLH) were involved in the development of the Standards. We acknowledge that this experience may have influenced their outlook and approach to the study. To mitigate potential biases, SM was the lead analyst to help maintain a more objective and independent perspective.
Results<br>• A statement reflecting on whether you think you caught the key characteristics, and a good spread of participants would support its transferability here.<br><br>• Effectiveness: see previous comments. The definition is interesting given that the results suggested that there was not enough evidence to do this due to the early stage of the Standards rollout. Some may also argue that measuring effectiveness requires a mixed methods or quantitative approach. There are valuable findings, however, on how participants were planning to map and measure the impact of the standards. You may wish to re-consider the definition of effectiveness, or add some reflection on this in the discussion?	We have strengthened the section at the beginning of the results section to clarify the different healthcare professionals and the number of local authorities area represented by participants;<br><br>HCPs (n=19, 26.0%) (GPs [n=6], nurses [=2], occupational therapists [n=1] and physiotherapists [n=10]) and policy stakeholders (n=11, 15.1%). The participants were drawn from 28 of the 32 local authority areas in Scotland, indicating a good spread of stakeholder groups and geographic representation.

	Please see our comment above – we have redefined the definition of effectiveness to still include these valuable findings.<br><br>As the paper is about implementation of the Standards, rather than implementation of PARS in general, we have not followed this through into the discussion, but have added into the new limitations section.
Discussion/conclusion<br>• The Principal findings state "We analysed stakeholder views…considering the reach, adoption, and implementation elements of the RE-AIM framework", should 'Effectiveness' be included as this was a category/theme in the findings?<br>• In the Principal findings it would be useful to emphasise the difficulty assessing effectiveness due to the early stage of the Standards, also see earlier comment on Effectiveness in the Results section.<br><br>• The assessment of inequalities and provision of PARS to marginalised groups seemed to be a common theme in the 'Effectiveness' Results section, participants wanting more guidance on addressing equality issues. Including this in the discussion would seem important.<br><br>• I am not sure where Implication number 5 "to explore how credibility of PARS can be enhanced in health collaborations" is supported in the findings, this may need a little explanation.	The first sentence of discussion has been amended to add in effectiveness.<br><br>Please see earlier comments<br><br>Thank you for this comment. We have added in the following to the discussion section: Participants in our study suggested a need for guidance to ensure that PARS successfully targeted health inequalities. The Standards highlight this as an area for attention, without giving specific guidance. Strategies that facilitate a reduction of health

	inequalities must be intersectoral and multidisciplinary in nature (42). As such, PARS must act as part of a wider system to provide great investment in service to support deprived communities (43) and currently underserved communities such as ethnic minority groups (44).<br><br>We have added clarification into the Implementation section:<br>PARS providers had differing opinions about whether implementing the Standards would help to build partnerships but felt that they provided focus and enhanced credibility, especially in health collaborations as this was considered to be limited at present.
References<br>• The reference for Framework approach appears to be about a tool to assess the quality of qualitative evaluations, not the Framework approach process, such as the commonly cited: Gale, NK et al (2013): http://www.biomedcentral.com/1471-2288/13/117	Thank you, we have added the suggested reference.
Reviewer: 3<br>Dr. Joanna Kesten, University of Bristol<br>Comments to the Author:<br>Thank you for the opportunity to review this interesting manuscript seeking to evaluate the initial rollout of the Physical Activity Referral Standards policy in Scotland. Overall, I think the reporting is comprehensive and clear. I have highlighted a few areas which I believe could be more transparently reported, particularly in relation to the sample's involvement and familiarity with the Standards, the	Thank you for your positive feedback.

analysis approach and consideration of the study’s methodological strengths and limitations.	
Study design<br>Could the authors add a little information on the selection of qualitative methods (as opposed to a mixed methods approach) to address the aim of this research? Is this included in the wider project which is referred to?	Yes, this study part of a larger funded project. The aim in this first part was to establish a baseline for aware of the Standards and intention to use. The part (currently underway) includes 1) a national survey of the design and delivery PARS, 2) collation of an anonymised dataset where PARS are collecting any aspects of the minimum national data set and 3) focus groups with participants to understand their views of how schemes should be monitored and evaluated. This means that overall it will be a mixed methods project, but we feel that this initial study is distinct and worthy of separate publication.<br>We have added in a shorter statement in the study design section to reflect the above:<br><br>This study was the first part of a larger project that includes a mixed methods approach to explore the design and
Sample<br>Please expand on the description of the purposive sampling approach. Which stakeholder characteristics/roles or experiences were you seeking?<br><br>Similarly, what were the inclusion / exclusion criteria for the study? Were any of the interviewees involved in the development or roll out of the Standards, for example? This is hinted at in the data collection and discussion sections but could be more transparently reported and reflected on in a strengths and limitations section (see comments below).<br><br>For qualitative research you interviewed a large sample. Please include an explanation about how you decided when to stop data collection.	

Results<br>The findings are mostly clearly reported and are valuable in understanding the early implementation of the Standards.<br><br>Please clarify, how was 'awareness of the standards' defined as 'high'? Does this reflect involvement in developing the standards (see earlier comment)? This is noted in the discussion but could be reported in the findings as an explanation, particularly if it is evident in the data.<br><br>Please explain what tiered models of PA interventions means for unfamiliar audience.<br><br>As above, please expand on what a 'minimum national data set' is for an unfamiliar audience.	delivery PARS, monitoring and evaluation strategies and participants views of how schemes should be monitored and evaluated.<br><br>Thank you for this comment (which is similar to reviewer 2): We have added more detail in the section describing the stakeholder roles:<br>A purposive sample of Scottish PARS stakeholders, including senior managers of PARS provider organisations (responsible for strategic planning for health and wellbeing activities), PARS coordinators (responsible for managing the day-to-day delivery of schemes), HCPs able to refer to PARS and those involved in developing and/or implementing PA policy (working in government, national sporting bodies, and Scottish NHS health boards). Within these groups, there were no exclusion criteria.<br><br>We have provided further clarify about the stakeholder pool. Within this group, there were no

	exclusion criteria.<br>We have added:<br>Within these groups, there were no exclusion criteria.<br><br>We have added the following:<br>The sample included stakeholders who had been involved in the Standards development.<br><br>Thank you for this comment, we have added the following explanation into our sample section:<br>Our sample included four stakeholder groups, and the sample size was based on the potential to achieve data saturation within each group at between 9 and 17 interviews (19). PARS coordinators were an exception, where a large sample size was required due to the variety of different approaches to scheme delivery.<br><br>Thank you for this positive comment.<br><br>We have added the following statement at the beginning of the reach section:<br>We considered level of >70% for a

	stakeholder group to be high.<br><br>We have added more detail to the following sentence in the results section:<br>The tiered model of PA interventions (a conceptual diagram indicating the target audience for interventions, the type of behaviour change approach, and levels of interventions that offer appropriate support for long-term conditions and readiness to change) (supplementary file 3)<br><br>We have clarified what the minimum data set is in the abstract:<br><br>Views about the minimum dataset (suggested essential or desirable data fields to be collected for monitoring and evaluation)<br><br>And have added in further detail at the end of the introduction to read:<br>… a national minimum data set, which included suggested essential or desirable data fields to be collected for monitoring and

	evaluation such as demographics, referral numbers, uptake and adherence)
Strengths and limitations<br>Please add a 'strengths and limitations' section to the discussion section. As above, one point to consider is whether there is a potential bias in the sample towards those who are aware and supportive of the standards? How likely is it that people who did not know about the standards at all would take part? What did you do to mitigate against this? In addition, please reflect on the possible impacts (including beneficial) of the study authors involvement in the development of the Standards (e.g. including the interpretation of the data). Did participants know about the study authors involvement in the development of the Standards? If so, what efforts were made to mitigate against social desirability bias?<br><br>Comparison to previous literature – it may also be useful to situate your findings in the context of the systems based literature. E.g. https://bmcmedresmethodol.biomedcentral.com/articles/10.1186/s12874-022-01570-4	Thank you for your suggestion. We have added in the following limitations section:<br><br>This study included a large sample to ensure that the voices of different Stakeholder groups were heard. We recognise that those who were aware and supportive of the Standards may have been more likely to take part. This was especially true for those involved in the Standards development. However, we mitigated against this by conducting internet searches to identify all PARS in Scotland and approached every scheme that we could find to invite them to participate. Two of the study authors were also involved in the Standards development and this may have influenced their interpretation of data. However, the lead analyst was not involved and was able to offer a more objective and

	independent perspective. Participants were not informed of author involvement in the Standards and the majority of interviews were not done by those involved. Some participants will have been aware, and this may have influenced answers.<br><br>Thank you for this comment. Due to the amount of extra text added because of other suggestions, we have not actioned this suggestion.
Abstract<br>In the abstract and main text please provide more detail on healthcare professionals involved in PARS (e.g. primary care, secondary care, GPs, nurses).<br><br>Participants: You could state the numbers from each stakeholder group (scheme providers, healthcare professionals, policy stakeholders) in the abstract to give an idea of the spread of the data.<br>Setting: you could include details about whether the sample included stakeholders from urban and rural areas.<br><br>Findings: A brief explanation of what a minimum dataset is for an unfamiliar audience would be beneficial.	Thank you for these suggestions. We have amended the abstract as follows:<br>**Participants:** 73 stakeholders including scheme managers (n=34), senior managers from provider organisations (n=9), healthcare professionals (n=19) (GPs, nurses, occupational therapists and physiotherapists), and policy stakeholders (n=11).<br><br>**Setting**: Data were collected across 28 local authority areas in rural and urban areas of Scotland between December 2022 and June 2023.

	Views about the minimum dataset (suggested essential or desirable data fields to be collected for monitoring and evaluation) contained in the Standards were divided.
Introduction<br>The introduction is clear and comprehensive.<br><br>I wonder if it would also be helpful to expand a little on the mechanisms through which PARS are expected to work, potentially referring to or presenting a logic model?<br><br>In addition, I note that the National Centre for Sport and Exercise Medicine has worked with partners to produce a toolkit for the design, implementation and evaluation of exercise and referral schemes. Would it be helpful to cite this toolkit and how it sits alongside the Public Health Scotland Standards? https://ncsem-em.org.uk/wp-content/uploads/2020/10/section-8-evaluating-exercise-referral-schemes.pdf<br><br>The final paragraph touches on the translational gap between evidence and practice. To expand on this and strengthen the rationale for this research, I wonder whether it would be useful to draw on literature around knowledge mobilisation, implementation science or theories such as Normalization Process Theory. RE-AIM is used in the analysis, describing this framework (and/or other literature) in the introduction could help explain the importance of understanding implementation processes to enhance delivery and uptake.	Thank you for this suggestion. We have included the logic model from the PARS Standards as a supplementary file and have referred to this in the introduction.<br><br>Thank you for this suggestion. The toolkit is a little outdated now but is an important part of policy/standards development for PARS. We have added the following to the introduction: In 2010 the British Heart Foundation National Centre for Physical Activity and Health published 'A Tool Kit for the Design, Implementation & Evaluation of Exercise Referral Schemes' (14). This suggested the need for PARS to create logic models and conduct process and outcome

	evaluations. The toolkit highlighted validated questionnaires previously used in PARS literature without specify usage preferences.<br><br>Due to word limitations and the number of other changes that we have made, we have not mentioned implementation science or other knowledge mobilisation literature.<br><br>We have, however, added in a sentence about RE-AIM to introduce it to the reader at this point. The RE-AIM framework is a commonly used planning and evaluation frameworks across public health, behaviour science and implementation science (19). This study aimed to understand the early policy delivery process for the Standards to inform ongoing implementation. We used RE-AIM as a framework in which to situate our findings and help explain whether and/or how the Standards are used.

PPI<br>Please can you clarify what is meant by a PARS participant – are these patients who have been referred to PAR?<br><br>It would be great to know a little more about the ways in which this involvement shaped the study methods and findings.	The study is part of a wider project with a steering committee that includes five PARS participants (people who had been referred to a PARS in Scotland).
Data collection<br>There is a good level of detail in this section.<br><br>It could also be worth clarifying (in line with the topic guides) what information was provided to the participants about the Standards / PARS particularly if their responses indicated they were not (very) aware of them?<br><br>Analysis<br>Both thematic analysis and framework analysis are employed. Can the authors provide a rationale for the inclusion of both approaches please?	All participants were provided with a link to the Standards prior to interviews (although this did not mean that they read them). We have added this in at the end of the sample paragraph<br>One week before interviews took place, participants were sent a link to the Standards so that they could read them if they wished to.<br><br>We have clarified our approach in the methods section, as this was raised by all reviewers:<br>“Interviews were transcribed verbatim, anonymised, and imported into NVivo20, a data analysis organisation tool. Data were analysed using the Framework Method (20). During the familiarisation phase, SM and CLH checked transcripts with audio

My primary concern about the analysis and reporting is that reclassifying the inductive themes into RE-AIM means they not apparent in the reporting. Could the authors consider presenting inductive themes as subthemes or more clearly illustrating how the initial inductive themes fit within RE-AIM?<br><br>The results state: “Enablers and barriers for the Standards rollout were mapped within the reach, effectiveness, adoption, and implementation elements of RE-AIM.” I wonder if this sentence fits better in the data collection or analysis section which do not mention enablers and barrier questions/themes/codes?<br><br>The analysis also does not mention the quantification of the findings presented in the results section.	recordings for accuracy. They independently created inductive open codes (n=97) for three transcripts in each stakeholder group. In the identification phase they grouped open codes into 12 initial inductive themes (awareness, usefulness, the tiered model of PA interventions, local delivery contexts, cost, inequalities, sharing good practice, workforce issues, data systems, monitoring/evaluation, communication, and partnerships). Initial themes were mapped to the reach, adoption, and implementation elements of the RE-AIM framework (21, 22) to structure the findings. This initial framework was applied as a tool for interpretation, as opposed to theme development, and presented to the other researchers during a data workshop. After a critical discussion, the inductive themes were reorganised within the RE-AIM framework to include effectiveness. During indexing, SM

	coded 10 more transcripts, which CLH checked for coding accuracy by reading code content and using NVIVO20 deductive word searching. SM then coded all remaining transcripts. NVivo framework matrices were used during the charting phase, (example supplementary file X). Researchers explored and rearranged the data within the inductive themes and compared across cases to develop explanatory summaries. Prior to final analysis, the framework was reviewed, and the analysis mapped to the reach, effectiveness, adoption, and implementation elements of RE-AIM."<br><br>Thank you for this suggestion. We have added a supplementary file that shows the coding trees for this aspect of the analysis. We have added in a reference to the supplementary file in the data analysis section.

	We agree that this sentence was not ideal and have changed it to:<br><br>Findings were mapped within the reach, effectiveness, adoption, and implementation elements of RE-AIM<br><br><br>Thank you for pointing out this omission. We have added the following: Where data were quantifiable (e.g., awareness of the Standards), we collated results in a Microsoft Excel Spreadsheet (Microsoft Corporation, St. Redmond, WA, USA),
Discussion<br>Implications<br>The implications of this work are valuable for supporting the adoption of the Standards.<br><br>Could the authors describe if any work has been done (or is planned) to mobilise this evidence and enhance delivery of the standards in partnership with key stakeholder groups please?	 <br>Thank you for this positive comment.<br><br><br>As previously commented, this is part of a larger study, and we are currently in the process of evaluating PARS mapping and data collection quality. We recently held a knowledge exchange event attended by 125 people, including representatives from Public Health Scotland, Scottish

	government, and many PARS providers. As a result, work is underway to create an online national PARS repository for sharing of learning and good practice, and to produce systems to support future monitoring and evaluation. We hope to publish this work in the coming year.
Strengths and limitations of this study<br>Please also consider whether the views captured may reflect more positive perceptions of the Standards and greater awareness of them than among those who did not participate? What did the team do to mitigate against this (see comment on this below too)?	We have added in a new limitations section:<br><br>This study included a large sample to ensure that the voices of different Stakeholder groups were heard. We recognise that those who were aware and supportive of the Standards may have been more likely to take part. This was especially true for those involved in the Standards development. However, we mitigated against this by conducting internet searches to identify all PARS in Scotland and approached every scheme that we could find to invite them to participate. Two of the study authors were also involved in the Standards development and

	this may have influenced their interpretation of data. However, the lead analyst was not involved and was able to offer a more objective and independent perspective. Participants were not informed of author involvement in the Standards and the majority of interviews were not done by those involved. Some participants will have been aware, and this may have influenced answers.

VERSION 2 - REVIEW

Reviewer 3

Name Kesten, Joanna

Affiliation University of Bristol, NIHR CLAHRC West and NIHR Health Protection Research Unit in Evaluation of Interventions

Date 18-Dec-2024

COI

Many thanks for engaging with all the reviewers comments so thoroughly and positively. The manuscript has been much strengthened as a result. I only have a small number of minor comments remaining. I hope these final points are helpful.

In my view, the description of data saturation needs further clarification and the interpretation of the level of knowledge and reach needs more consideration.

Minor typos: 'great' should be 'greater': PARS must act as part of a wider system to provider great.

'Specify' should be 'specific': The toolkit highlighted validated…

Missing the word ‘more’: This involved the development of a logic model (supplementary file 1) that focused on the wider PARS system ‘more’ than the logic model developed for the 2010 Toolkit.

Thank you for clarifying that you were working to data saturation to determine the sample size. Please can you add a little more detail on how you assessed whether you achieved this (e.g. analysing the data alongside data collection) and whether 9 to 17 interviews is based on previous research? Similarly, was a large sample of PARS coordinators predetermined or required to reach data saturation?

The description of the assessment of level of knowledge is still unclear to me “We considered level of >70% for a stakeholder group to be high.” Is a word missing? Does this relate to the question in the topic guide: Were you aware of the PHS PAR Standards prior to this study? Does high knowledge mean participants answered yes to this question? If so, I’m not convinced, that ‘high’ is an accurate term to reflect this data as they may have heard of it but not know much about it. I think this is important because it relates to the conclusions around ‘high reach’ of the Standards. Given this is a qualitative study and the finding that healthcare professionals were perceived to lack awareness of the standards, I’m not sure that firm conclusions about reach (as with the reviewer comments about effectiveness) can be made.

VERSION 2 - AUTHOR RESPONSE

Reviewer comment	Response
Many thanks for engaging with all the reviewers comments so thoroughly and positively. The manuscript has been much strengthened as a result. I only have a small number of minor comments remaining. I hope these final points are helpful.<br><br>In my view, the description of data saturation needs further clarification and the interpretation of the level of knowledge and reach needs more consideration.	Thank you for this positive feedback. We have now made some final amendments based on your comments.<br><br>We have addressed these in the sections below.
Minor typos:<br><br>‘great’ should be ‘greater’: PARS must act as part of a wider system to provider great.<br><br>‘Specify’ should be ‘specific’: The toolkit highlighted validated...	Thank you for pointing out these errors. We have corrected them all<br><br>PARS must act as part of a wider system to provide greater investment in services to support deprived communities

Missing the word 'more': This involved the development of a logic model (supplementary file 1) that focused on the wider PARS system 'more' than the logic model developed for the 2010 Toolkit.	The toolkit highlighted validated questionnaires previously used in PARS literature without specific usage preferences.<br><br>This involved the development of a logic model (supplementary file 1) that focused on the wider PARS system more than the logic model developed for the 2010 Toolkit.
Thank you for clarifying that you were working to data saturation to determine the sample size. Please can you add a little more detail on how you assessed whether you achieved this (e.g. analysing the data alongside data collection) and whether 9 to 17 interviews is based on previous research?<br><br>Similarly, was a large sample of PARS coordinators predetermined or required to reach data saturation?	We have added in more detail as requested: between 9 and 17 interviews, based on systematic review recommendations by Hennink and Kaiser.[20]<br><br>In the analysis section we have added: Analysis took place alongside data collection to gauge when no new meaning of codes or themes and their relationships were apparent.<br><br>We had already highlighted the reason for this:<br>PARS coordinators were an exception, where a large sample size was required due to the variety of different approaches to scheme delivery.<br><br>Please note that we had a much larger response from this group than we expected, even given the variety of approaches.
The description of the assessment of level of knowledge is still unclear to me "We considered level of >70% for a stakeholder group to be high." Is a word missing? Does this relate to the question in the topic guide: Were you aware of the PHS PAR Standards prior to this study? Does high knowledge mean participants answered yes to this question? If so, I'm not convinced, that 'high' is an accurate term to reflect this data as they may have heard of it but not know much about it. I think this is important because it relates to the conclusions around 'high reach' of the Standards. Given this is a qualitative study and the finding that healthcare professionals were perceived to lack awareness of the standards, I'm not sure that firm conclusions about reach (as with the reviewer comments about effectiveness) can be made	Thank you for bringing this to our attention. We agreed that awareness does not relate to in level of knowledge and so we have changed this to read:<br><br>Overall, 72.6% (n=53) of stakeholders were aware that the Standards existed prior to the study but this varied by stakeholder group; PARS provider senior managers (n=8/9, 88.9%), PARS coordinators (n=27/34, 79.9%), policy stakeholders (n=11/11 100%) and HCPs (n=7/18, 36.8%). However, level of knowledge about content varied, with policy stakeholders and PARS coordinators being able to describe/discuss more components without prompting during interviews.<br><br>We have also changed the principal finding to state:<br>Seventy three percent of participants were aware of the Standards (reach), although this

	varied between stakeholder groups and HCPs were the least informed.
